# Review of the Delivery Kinetics of Thermosensitive Liposomes

**DOI:** 10.3390/cancers15020398

**Published:** 2023-01-07

**Authors:** Dieter Haemmerich, Krishna K. Ramajayam, Danforth A. Newton

**Affiliations:** 1 Department of Pediatrics, Medical University of South Carolina, Charleston, SC 29425, USA; 2 Department of Bioengineering, Clemson University, Clemson, SC 29634, USA

**Keywords:** thermosensitive liposomes, hyperthermia, cancer, nanoparticles, drug delivery systems, chemotherapy

## Abstract

**Simple Summary:**

Various nanoparticles have been developed over the last few decades for targeted drug delivery to cancerous tumors while reducing toxicities. Thermosensitive liposomes (TSL) belong to the category of triggered nanoparticle delivery systems, where drug release occurs in response to hyperthermic temperatures (typically, >40 ºC). After administration, the TSL-encapsulated drug circulates for extended duration (hours) in the blood stream. Localized hyperthermia of the targeted tissue results in localized drug release, enabling up to 25x tumor drug uptake compared to administration of unencapsulated drug. Here, we review the delivery kinetics of TSL and discuss how the interaction between drug, TSL and hyperthermia device affects drug delivery. Thus, this review provides guidelines on how to improve drug delivery by optimizing the combination of TSL, drug, and hyperthermia method. Many of the concepts discussed are applicable to a variety of other triggered nanoparticle delivery systems.

**Abstract:**

Thermosensitive liposomes (TSL) are triggered nanoparticles that release the encapsulated drug in response to hyperthermia. Combined with localized hyperthermia, TSL enabled loco-regional drug delivery to tumors with reduced systemic toxicities. More recent TSL formulations are based on intravascular triggered release, where drug release occurs within the microvasculature. Thus, this delivery strategy does not require enhanced permeability and retention (EPR). Compared to traditional nanoparticle drug delivery systems based on EPR with passive or active tumor targeting (typically <5%ID/g tumor), TSL can achieve superior tumor drug uptake (>10%ID/g tumor). Numerous TSL formulations have been combined with various drugs and hyperthermia devices in preclinical and clinical studies over the last four decades. Here, we review how the properties of TSL dictate delivery and discuss the advantages of rapid drug release from TSL. We show the benefits of selecting a drug with rapid extraction by tissue, and with quick cellular uptake. Furthermore, the optimal characteristics of hyperthermia devices are reviewed, and impact of tumor biology and cancer cell characteristics are discussed. Thus, this review provides guidelines on how to improve drug delivery with TSL by optimizing the combination of TSL, drug, and hyperthermia method. Many of the concepts discussed are applicable to a variety of other triggered drug delivery systems.

## 1. Introduction

**Figure 1:**
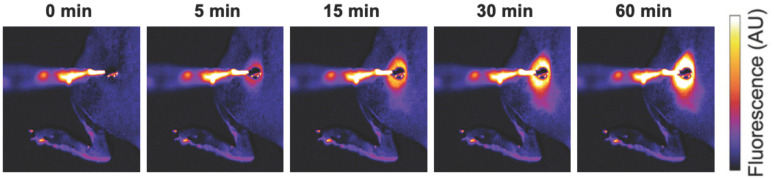
Localized drug delivery with thermosensitive liposomes (TSL). Following the administration of TSL-encapsulated doxorubicin (Dox), a subcutaneous mouse tumor was heated by a surface heating probe to 43 °C. Fluorescence imaging during hyperthermia visualizes the localized delivery of the fluorescent drug (Dox). Drug delivery takes place as long as hyperthermia is applied, here visualized by a fluorescence increase over the 60 min heating duration. Figure reproduced from [30] (published under Creative Commons CC BY license).

Thermosensitive liposomes (TSL) belong to the category of triggered nanoparticle drug delivery systems (DDS) where a drug associated with the DDS is released in response to an external trigger [1,2,3,4]. TSL are triggered by heat and release the encapsulated drug when exposed to mild hyperthermia (HT), typically ~40–43 °C. TSL were first described more than four decades ago [5,6,7,8]. Since then, numerous TSL formulations combined with various drugs have been described, as summarized in prior reviews [9,10,11,12,13,14,15]. TSL are most often administered systemically, e.g., by intravenous infusion, and then circulate in the blood stream for an extended duration. Combined with localized hyperthermia, TSL enable loco-regional drug delivery (Figure 1). This enables the delivery of a large drug dose to a targeted tissue region (e.g., tumor) while reducing systemic toxicities. Therefore, TSL are attractive as a therapeutic strategy in cancer patients where loco-regional drug delivery is beneficial, but less useful in metastatic cancer patients that require systemic therapy. While TSL have been most widely investigated for drug delivery in cancer therapy, additional potential clinical applications include the delivery of antibiotics [16,17], the treatment of inflammatory diseases [18], and the treatment of blood clots [19].

**Figure 2:**
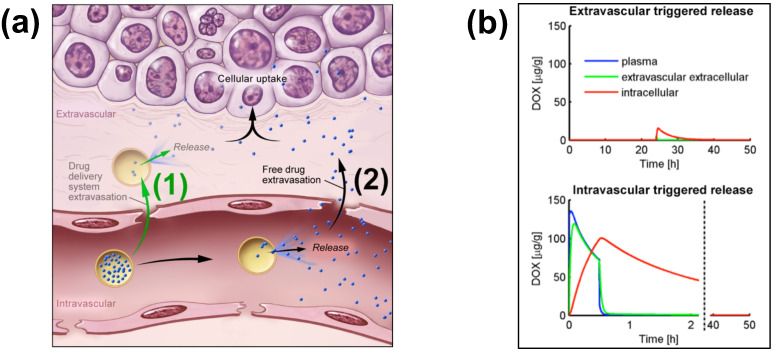
Extra- and Intra-vascular triggered release. (**a**) (1) Traditionally, nanoparticle DDS have been based on passive tumor targeting due to enhanced permeability and retention (EPR), where drug is released following extravasation of the DDS. (2) For TSL with intravascular triggered release, EPR is not relevant: TSL enter the tumor microvasculature of the target region where the release trigger (i.e., hyperthermia) is present, and release the contained drug within the vasculature. The released drug extravasates rapidly into tissue and is then taken up by cancer cells. (**b**) *Top graph:* Concentration dynamics in plasma, interstitial, and intracellular compartments during extravascular triggered release. TSL were allowed to accumulate for 24 h in the tumor based on EPR, followed by hyperthermia triggered release. *Bottom graph:* Concentration dynamics during intravascular triggered release. Hyperthermia (30 min) was applied immediately after TSL administration. Concentration increases in plasma due to drug release. Released drug then extravasates into interstitium (extravascular extracellular space), where it is taken up by cells. Figure 3a reproduced from [20] (published under CC BY 4.0 license). Figure 3b reproduced from [22] (published under CC0 license).

TSL enable two different delivery approaches: extravascular triggered release, and intravascular triggered release (Figure 2) [14,20,21,22,23]. Extravascular triggered release requires the extravasation of the TSL, followed by HT-triggered release of the encapsulated agent [14,24]. This extravasation is based on TSL accumulation within the tumor interstitium facilitated by enhanced permeability and retention (EPR) [14,25,26,27,28]. Several recent papers described the limitations of the EPR effect, such as high intra- and inter-tumor variability, and an apparent upper delivery limit [14,27,29]. Recent reviews highlight the need for delivery strategies that do not rely on EPR [14,27,29].

**Figure 3:**
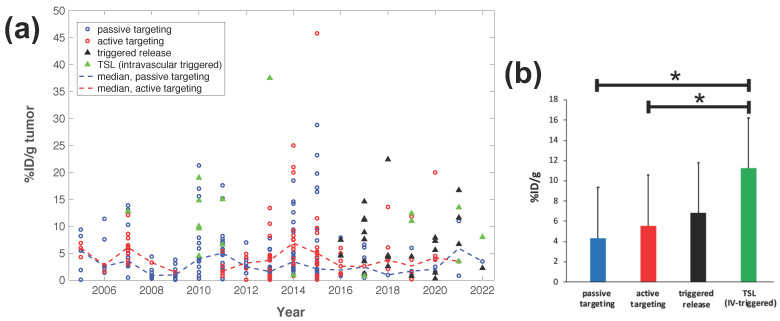
Delivery efficacy of intravascular triggered TSL compared to other nanoparticle DDS. A prior review compared the efficacy of 117 nanoparticle DDS studies published between 2005–2015 [27], and we combined data from this prior review to include studies published between 2016–2022 based on the same search algorithm [30,31,32,34,35,36,37,38,39,40,41,42,43,44,45,46,47,48,49,50,51,52,53,54,55,56,57,58,59,60,61,62,63,64,65,66,67,68,69,70,71,72,73,74,75,76,77,78,79,80,81,82,83,84,85,86,87,88,89,90,91,92,93,94,95,96,97,98,99,100,101,102,103,104,105,106,107,108,109,110,111,112,113,114]. (**a**) Plot showing the delivery efficacy (%injected dose per gram tumor (%ID/g tumor)) based on the combined data [27,30,31,32,34,35,36,37,38,39,40,41,42,43,44,45,46,47,48,49,50,51,52,53,54,55,56,57,58,59,60,61,62,63,64,65,66,67,68,69,70,71,72,73,74,75,76,77,78,79,80,81,82,83,84,85,86,87,88,89,90,91,92,93,94,95,96,97,98,99,100,101,102,103,104,105,106,107,108,109,110,111,112,113,114]. Each marker represents a published study, and dashed lines indicate the annual median for DDS with passive and active targeting. (**b**) The means of all prior studies in each category between 2005–2022 are compared, suggesting superior delivery efficacy of intravascular triggered TSL (* indicates statistical significance (*p* < 0.05)).

Intravascular triggered release is a strategy where drug release occurs in the microvasculature while the TSL pass through the heated tumor, and does not require the EPR effect (Figure 2a) [6,20,21,22,23]. Many of the more recent TSL formulations are based on intravascular triggered release, and such TSL have demonstrated superior delivery efficacy, with up to 25× higher drug delivery compared to unencapsulated drugs [31]. Compared to non-triggered nanoparticle drug delivery systems, TSL based on intravascular triggered delivery demonstrate superior tumor drug uptake (Figure 3). In addition, the direct comparison of TSL with extra- versus intra-vascular triggered delivery strongly suggests that the latter is superior [22,24,32,33] (Figure 2b). Therefore, in the remainder of this review, we will focus on drug delivery by TSL via intravascular triggered release.

### Tissue Transit Time

**Figure 4:**
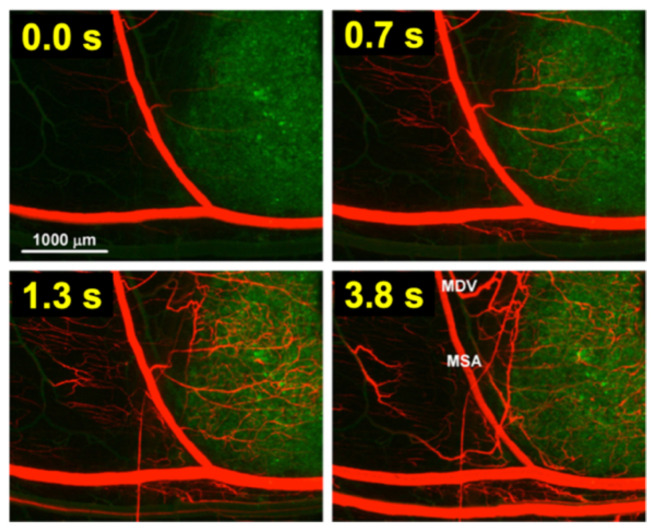
Tumor plasma transit time. Tumor (green fluorescent labeled cancer cells) was imaged by intravital fluorescence microscopy. A red fluorescent contrast agent was injected as bolus. The time at left upper corner of each image indicates timing relative to plasma first entering the tumor segment; plasma exits the tumor segment again within ~4 s (note: red blood cells move slower than plasma and remain longer in the tumor segment). In the final image (right lower corner), the main supplying artery (MSA), and main draining vein (MDV) of the imaged tumor segment are labeled. Figure reproduced with permission from [115].

For TSL based on intravascular triggered release, the dynamics of blood flow through the tumor vasculature is of primary relevance. Blood/plasma with TSL enter a tumor segment through a supplying artery, pass through tumor capillaries, and exit the tumor segment through a draining vein. The average time that plasma spends within a tumor segment is termed the ‘tissue transit time’ (*TT*) (compared to plasma, red blood cells move significantly slower through capillaries, and thus remain for longer within the tumor segment [115]). The drug release from TSL, and drug extraction by tumor tissue, can only occur during this tissue transit time. Figure 4 visualizes the transit time between supplying artery and draining vein of a small mouse tumor segment. In human tumors, the mean transit time through a tumor has been measured for various tumor types. This mean tumor transit time varies widely, and is ~2 s for primary hepatocellular carcinoma [116], ~3 s for head and neck and prostate tumors [117,118], ~11 s for renal cell carcinoma [119], ~25 s for metastases to the liver [116], and ~30 s for breast cancer [120]. Furthermore, transit time and perfusion vary spatially within tumors such that transit time can be locally within a tumor considerably higher or lower than these mean values that were averaged over the whole tumor.

**Figure 5:**
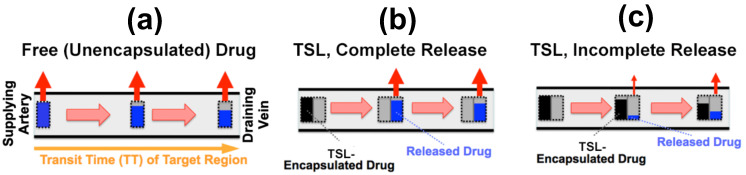
*Microvascular concentration gradient.* Plasma flows within a representative capillary between the supplying artery and draining vein of a tumor segment. Plasma concentration of unencapsulated/released drug (blue bar), and TSL-encapsulated drug (black bar) are shown, with red arrows indicating tumor drug uptake (i.e., drug extraction). Three cases are presented: (**a**) Unencapsulated drug infusion into supplying artery, (**b**) TSL with complete release during transit, and **c**) TSL with incomplete release during transit. In (**b**,**c**), drug is first released from TSL, followed by tissue uptake. Note that all figures show first pass where no drug is yet present in the tissue interstitium. Figure reproduced from [20] (published under CC BY 4.0 license).

As plasma with TSL enters capillary vessels within a heated tumor region, the TSL start releasing the drug and the drug is then extracted by the tumor (Figure 5b,c). Therefore, the plasma drug concentration varies along the vasculature as plasma flows between the supplying artery and draining vein of a tumor segment—in other words, a concentration gradient develops along the tumor microvasculature between the supplying artery and draining vein. Figure 5 illustrates schematically this microvascular concentration gradient along a representative capillary connecting the supplying artery and the draining vein of a tumor segment. For free (unencapsulated) drug, plasma drug concentration decreases as drug is extracted (Figure 5a). For TSL, drug is first released by hyperthermia, and then the released (free) drug is extracted (Figure 5b,c). Ideally, TSL completely release the encapsulated drug during the transit time to maximize tumor drug uptake—i.e., TSL that release their drug within seconds are preferable (Figure 5b).

## 2. Impact of TSL Properties on Drug Delivery

**Figure 6:**
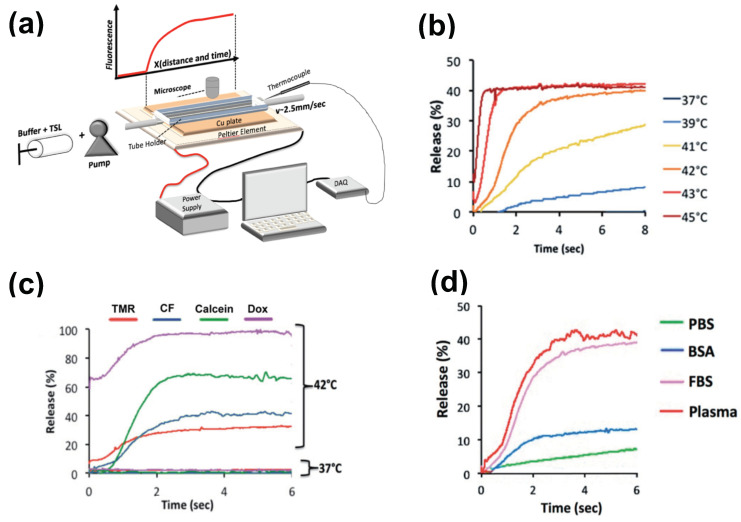
Measuring release kinetics of fast-release TSL formulations. (**a**) Millifluidic release assay schematics. A TSL solution (TSL + buffer) is pumped through a capillary tube that has been heated to the desired temperature by a Peltier element. Once the TSL solution enters the heated region, TSL begin to release the fluorescent drug/dye, resulting in a fluorescence gradient along the tube (upper graph). The Peltier temperature is measured by a thermocouple, and a control algorithm regulates the power applied to the Peltier element to control temperature. (**b**) Release of carboxyfluorescein (CF) from fast-releasing TSL (DPPC:MSPC:DSPE-PEG2000 = 85:10:5) between 37 and 45 °C during the first 8 s. Release within seconds is required to take advantage of the intra-vascular triggered release paradigm. (**c**) Release depends on the encapsulated compound, shown for four compounds for the same TSL formulation. Release in (**a**–**c**) was measured using fetal bovine serum (FBS) as buffer. (**d**) Release kinetics vary between buffers. CF release is shown for 4 buffers: phosphate buffered saline (PBS), 10% bovine serum albumin (BSA) solution, fetal bovine serum (FBS), and human plasma. TSL formulation used in (**b**–**d**) was identical. Figures reproduced with permission from [121].

The methods for preparation and loading of various TSL formulations with different agents has been reviewed extensively in prior reviews [9,10,11,12,13,14,15]. Additionally, the factors and mechanisms that affect drug release from TSL have been summarized in detail in earlier publications [9,11,12]. Here, we focus on reviewing how TSL properties such as release kinetics and plasma stability affect drug delivery. These properties depend both on the TSL formulation and the drug. For example, the same formulation will have varying release kinetics depending on which drug is encapsulated [121]. In addition, the buffer used to measure release affects release kinetics [121], highlighting the importance of selecting an appropriate buffer (e.g., plasma) (Figure 6d).

### 2.1. TSL Release Kinetics

The early TSL formulations had comparably slow release (within minutes to hours) [122,123]. In addition, heating to >42 °C was required to achieve substantial release. This is disadvantageous since temperatures above 43 °C may result in reduced blood flow [124] that would also reduce the inflow of TSL-encapsulated drug. The first fast-release TSL formulations were published in the early 1990s, demonstrating substantial release within a few seconds after heating to >41 °C [125,126]. Such rapid release is required to take full advantage of the intravascular triggered release paradigm, as discussed above. The first fast-release formulation with substantial release at lower temperatures (40 °C) was presented around the year 2000 [31,127,128], and formed the basis for the first commercial TSL formulation (ThermoDox®) that has been employed in several human clinical trials [129,130,131,132,133,134,135]. Several additional fast-release TSL formulations encapsulating various agents have been presented within the last two decades [136,137,138]. Recent studies confirm that fast-release TSL that release within a few seconds can deliver substantially higher drug amounts compared to slower releasing formulations [20,21,22]. However, for most of these TSL formulations, release kinetics is not known within the time scale relevant for intravascular triggered release (e.g., within the first few seconds), owing to limitations of conventional methods used for measuring the release kinetics.

The most widely used method for measuring TSL release kinetics employs a buffer pre-heated to the desired temperature, where a small volume of TSL is added, typically under stirring [113,123,137,139,140,141,142,143]. Release is quantified usually using spectrophotometry, since optical properties (e.g., fluorescence) change when the drug is released from TSL. Due to the time required for mixing of the TSL with the buffer, the first reported time points are typically between 8–20 s. This time is substantially longer than many typical tumor transit times, making these measurements of limited value.

There have been two methods presented to measure TSL release kinetics at short (second) time scales. The first method employed a small-diameter tube within which TSL solution was passed through heated water for a specific time, and the released drug was quantified in the sample exiting the tube [125]. An advantage of this method is that various quantification methods can be employed on these samples. In a second method, a glass capillary tube was heated by a Peltier element, and release was quantified by measuring fluorescence along the tube by either microscopic or macroscopic fluorescence imaging (Figure 6a) [121,144]. This method provides data at high temporal resolution not possible with other methods (Figure 6b–d), but is limited to fluorescent agents. In both methods, it is important to select thin-walled tubes and to validate sufficiently rapid heating of the solution passing though the tube to target temperature [121].

**Figure 7:**
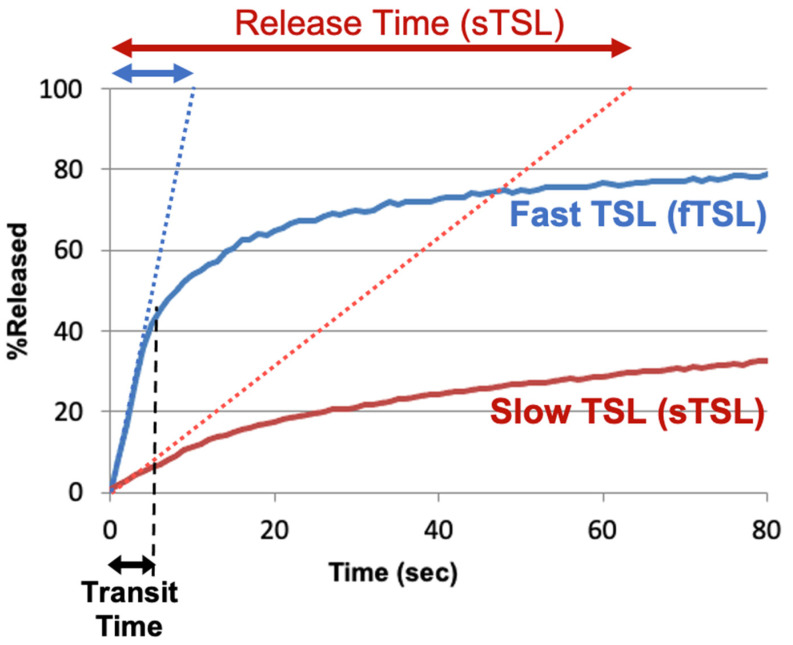
TSL release time. Release of two TSL formulations encapsulating a fluorescent drug analog (carboxyfluorescein) with slow (sTSL) and fast (fTSL) release is plotted, based on data from a prior study [20]. The dotted lines indicate a linear approximation of the release kinetics. TSL only spend a few seconds within the heated tumor (see black double arrow indicating ‘Transit Time’). Thus, in most cases, a linear approximation adequately represents release within those few seconds that TSL spend within the tumor vessels. Based on this linear approximation, a characteristic ‘release time’ is determined (indicated by red and blue double-arrows at the top) that enables the comparison of different TSL formulations. This release time was 8.2 s for fTSL, and 63.0 s for sTSL. The fraction of drug released during transit can be estimated by the ratio of transit time to release time (see Supplementary Material File S1, Equation (S2)).

**Figure 8:**
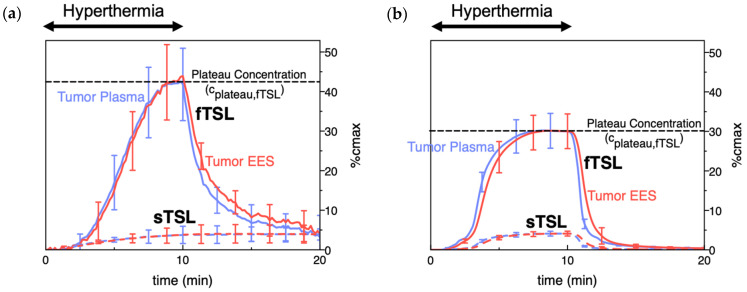
TSL delivery kinetics. (**a**) Drug concentration in plasma and interstitium (extracellular-extravascular space, EES) was determined from intravital microscopy data. Hyperthermia (42 °C) for 10 min was applied following the administration of either slow- (sTSL) or fast-release TSL (fTSL) encapsulating a fluorescent drug analog (carboxyfluorescein) (see Figure 7 for release kinetics of sTSL and fTSL). Plasma concentration increases during hyperthermia due to drug release. Released drug is then extracted by tissue, indicated by increasing interstitial (EES) concentration. A plateau (peak) concentration is approached towards the end of hyperthermia. This plateau concentration is substantially higher for fTSL compared to sTSL. Error bars indicate standard deviation (n = 3 animals/group). (**b**) Computer simulation of drug delivery kinetics based on in vivo measured tumor properties reproduces the delivery kinetics observed in (**a**). Error bars indicate computer model uncertainty due to uncertainty of model parameters. Figures reproduced from [20] (published under CC BY 4.0 license).

To compare the release kinetics of TSL formulations, a recent study suggested using a characteristic release time based on a linear approximation of the TSL release kinetics [20]. As noted earlier, TSL only spend a few seconds within a tumor (=transit time), and for most TSL, the release kinetics within those first seconds can be adequately represented by a linear approximation (Figure 7). Ideally, this release time would be smaller than the transit time to maximize release and tissue drug uptake (Figure 5). The amount of drug released during tumor transit (Figure 5) can be estimated by the ratio of transit time to release time (see Supplementary Material File S1, Equation (S2)). A recent study demonstrated that TSL with rapid release (i.e., short release time) can deliver substantially more drug to tissue than TSL with slow release (Figure 8) [20]. Table 1 summarizes the release times of published fast-release TSL formulations. In most cases, the exact release times could not be determined owing to limitations of methods used to quantify the release kinetics, as described above.

### 2.2. Plasma Stability

**Figure 9:**
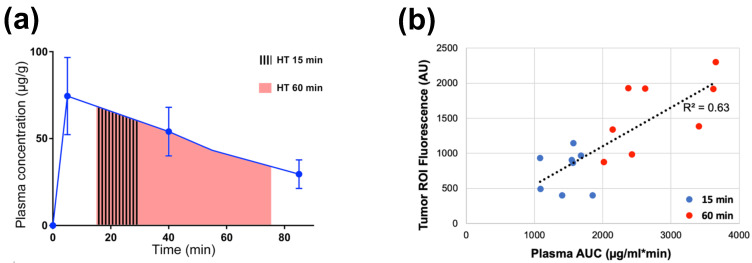
Plasma-AUC during hyperthermia correlates with tumor drug uptake. (**a**) AUC of plasma Dox concentration was calculated during heating, for either 15 or 60 min hyperthermia (HT) as indicated by shaded regions. (**b**) Plasma-AUC during HT correlated well with Dox fluorescence in the tumor region-of-interest measured following HT (R^2^ = 0.63). Tumors were exposed to hyperthermia (43 °C) for either 15 min (blue dots) or 60 min (red dots). Data reproduced from [162] (published under CC BY 4.0 license).

Plasma stability describes how long TSL-encapsulated drug remains in the systemic circulation after administration and can be quantified by the initial plasma half-life of a TSL formulation. Similar to the TSL release kinetics, plasma stability depends both on TSL formulation and encapsulated drug, but also varies with species (Table 1). During hyperthermia, circulating TSL-encapsulated drug continuously enters the heated tissue volume, with subsequent intravascular drug release (Figure 2 and Figure 5). The plasma concentration of TSL-encapsulated drug represents the amount available for intravascular triggered release. Thus, the AUC (area under the concentration vs. time curve) of the plasma concentration calculated during hyperthermia correlates with the total amount of TSL-encapsulated drug subjected to hyperthermia [161,162]. As a result, this AUC directly correlates with the amount of drug released in the heated tumor (see Supplementary Material File S1, Equation (S5)). This AUC also correlates with tumor drug uptake, as initially demonstrated in a computer modeling study [161] and later confirmed by several experimental studies (Figure 9) [149,162,163]. A higher plasma stability would therefore increase this AUC, resulting in larger amount of drug being released—assuming that the kinetics of TSL release is not different (e.g., increased plasma stability of a TSL formulation may be disadvantageous if it is associated with slower release). Similarly, one approach to enhance drug delivery is to adjust the timing of hyperthermia as to maximize the plasma AUC during heating [161,163]. 

Note however that such comparisons based on AUC are only appropriate for different studies with the same TSL formulation, and the same or similar hyperthermia methods (i.e., with similar tumor temperature). The AUC indicates the total amount of TSL-encapsulated drug that passes through the heated tissue during hyperthermia. If two different heating devices with different temperature profiles and heating volumes are used, the amount of drug released from TSL will differ. Similarly, if two different TSL formulations are used, the amount of drug released will differ due to varying TSL release kinetics. Thus, even if the AUC is identical (=total amount of TSL-encapsulated drug passing through heated tissue), the amount released from these two TSL formulations will vary, resulting in different tumor drug uptake.

TSL plasma stability depends on several factors, and one major contributor is drug leakage from TSL at body temperature (37 °C)—i.e., drug slowly leaks from TSL while in systemic circulation [164]. Unfortunately, the release rate at body temperature is usually tied to the release rate at hyperthermic temperatures—i.e., slow release at 37 °C and rapid release at hyperthermia represent conflicting requirements for TSL formulations.

The peak plasma concentration after administration of TSL-encapsulated drug (Figure 9a) naturally correlates with the administered dose. Often, the administered dose is close to, or at the maximum tolerated dose (MTD) for that particular TSL–drug formulation in the studied species. In rodents, the MTD relative to body weight is often substantially higher compared to humans [165]. This higher administered dose in rodents results in higher plasma concentration (Figure 9a) and higher tumor drug uptake compared to large animals [147,149,155] and humans [129,133]. This issue may be relevant when extrapolating results on tumor drug uptake and therapeutic response from rodent studies to human patients.

## 3. Impact of Drug Properties on Drug Delivery

In addition to TSL properties, the properties of the encapsulated drug have a major impact on drug delivery facilitated by TSL. As discussed earlier, both the TSL release kinetics and plasma stability are impacted by the selected drug (Figure 6c; Table 1). Thus, delivery is indirectly affected by the interaction between drug and TSL. In addition, the properties of the unencapsulated drug (i.e., once released) have a substantial impact.

### 3.1. Tissue Extraction (Vascular Permeability)

TSL based on the intravascular triggered release paradigm are equivalent to the direct infusion of unencapsulated drug into the tumor-feeding vessels (Figure 2 and Figure 5). This delivery paradigm is similar to intra-arterial drug infusion—a clinically used delivery strategy where the drug is directly infused into tumor-feeding vessels through catheters. It is well known that the drugs optimal for intra-arterial delivery are not necessarily identical to those optimal for systemic delivery, and that rapidly extracted drugs are preferable [166]. The parameter ‘extraction fraction’ (EF) (or ‘extraction ratio’) indicates the drug fraction extracted by tissue when infused via a supplying artery, during a single pass. This extraction fraction varies widely between drugs, and is in the range of ~0.2–1 for many chemotherapy agents (Table 2).

**Figure 10:**
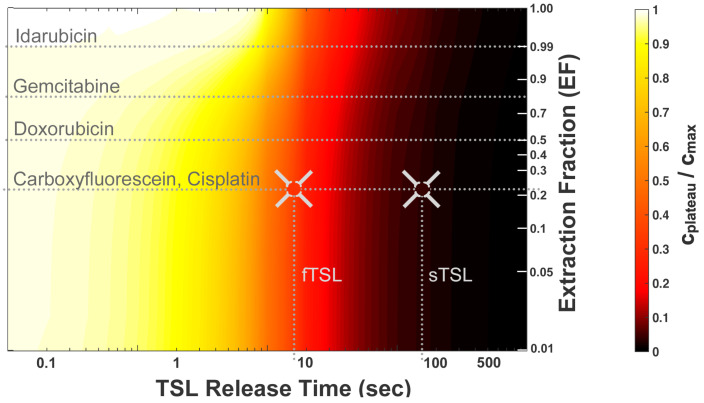
Drug extraction fraction (EF) and TSL release time dictate delivery efficacy. A parametric study was conducted based on an in vivo-validated computer model. The parametric study examined the interaction of two parameters that affect the maximum drug concentration achievable within the tissue interstitium (=plateau concentration (c_plateau_); see Figure 8): (1) TSL release time (see Figure 7), and (2) drug extraction fraction (EF). Delivery efficacy is indicated by the plateau concentration relative to maximum (c_plateau_/c_max_), and is visualized by a color scale. The dotted horizontal lines indicate EF for four common chemotherapy agents, and for a common fluorescent drug analog (carboxyfluorescein). In a prior in vivo study, carboxyfluorescein was encapsulated in two TSL formulations: a fast-release (fTSL) and a slow-release formulation (sTSL) (compare Figure 7). The two vertical dotted lines indicate the release times for fTSL (release time = 8.2 s) and for sTSL (release time = 63.0 s). The intersections of the horizontal dotted line corresponding to carboxyfluorescein and the vertical dotted lines indicate the location of experimental in vivo results for fTSL and sTSL in this map. The color inside the gray crosshairs corresponds to the measured in vivo plateau concentration. For highly permeable drugs (e.g., idarubicin, EF~1), a release time in the range of seconds is sufficient for near optimal delivery. For lower permeable drugs (e.g., cisplatin, EF~0.2), about 10× faster release (<0.1 s) is ideal. This study assumed a tumor transit time of 5.0 s based on in vivo measurements [20]. Figure reproduced from [20] (published under CC BY 4.0 license).

A recent study evaluated how tumor drug uptake varies depending on drug extraction fraction (EF), concluding that drugs with high extraction fraction are preferable for triggered DDS such as TSL [20]—similar to intra-arterial drug infusion [166]. In addition, the EF of the selected drug determines optimal TSL release kinetics (i.e., release time) for maximum delivery. For highly permeable drugs that are completely extracted (EF~1), a release time equal to or below the tumor transit time is sufficient. For drugs with lower EF, a much more rapid release time of less than 10% of transit time (release time << 1 s) is ideal (Figure 10). This may explain why prior studies with TSL-encapsulated cisplatin—a drug with comparably low EF (Table 2), that is, in addition, taken up slowly by cells [171]—has achieved limited delivery efficacy. Prior studies demonstrated a limited drug uptake enhancement of 2- to 4-fold (or ~1–5%ID/g) for TSL–cisplatin combined with hyperthermia, compared to control animals where free cisplatin was administered with hyperthermia [113,114,125,159]. 

### 3.2. Cell Uptake Kinetics

**Figure 11:**
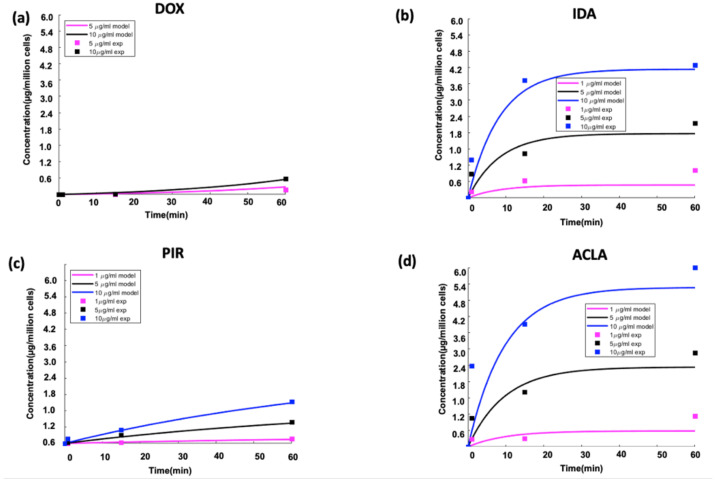
Cell uptake kinetics depend on drug. In vitro drug uptake of SVR (angiosarcoma) cells for (**a**) doxorubicin (DOX), (**b**) idarubicin (IDA), (**c**) pirarubicin (PIR), and (**d**) aclarubicin (ACLA) at extracellular concentrations of 1, 5 and 10 µg/mL. Uptake is shown for 60 min, which is a typical time used for hyperthermia-triggered delivery by TSL. Figure reproduced from [172] (published under CC BY license).

**Figure 12:**
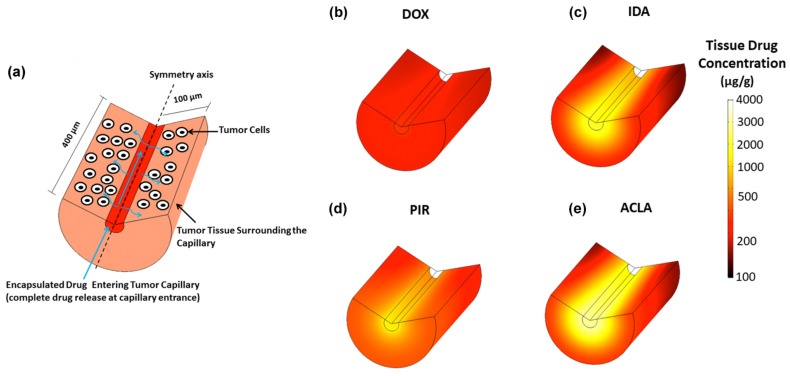
TSL delivery depends on drug. (**a**) Computer model simulated TSL-based drug delivery to SVR cancer cells surrounding a tumor capillary. Drug concentration after 60 min hyperthermia is shown for (**b**) doxorubicin (DOX), (**c**) idarubicin (IDA), (**d**) pirarubicin (PIR), and (**e**) aclarubicin (ACLA). Tumor drug uptake in (**b**–**e**) is largely dictated by cell uptake kinetics of the drug (see Figure 11). Drugs with fast cell uptake (ACLA, IDA) also result in highest tissue uptake. Figure reproduced from [172] (published under CC BY license).

During drug delivery with TSL based on the intravascular triggered release paradigm, the drug is released from the TSL within the vasculature and then diffuses across the vessel wall into the interstitial space (Figure 2 and Figure 5). This interstitial drug is then available for cellular uptake. The cell uptake kinetics depend on the drug (Figure 11), and may also vary with cell type. One recent study compared two anthracycline chemotherapy agents (doxorubicin and idarubicin) in mouse tumors. In vitro experiments in this prior study indicated much more rapid cell uptake of idarubicin compared to doxorubicin, which presumably contributed to the much higher tumor uptake of idarubicin [105]. Another recent study evaluated four anthracycline chemotherapy agents in computer models, where cell uptake kinetics were based on in vitro experiments with each drug in three cancer cell lines [172]. This study demonstrated that tumor drug uptake is significantly affected by cell uptake kinetics, and that drugs with rapid cell uptake (Figure 11) resulted in highest tumor drug uptake (Figure 12).

**Figure 13:**
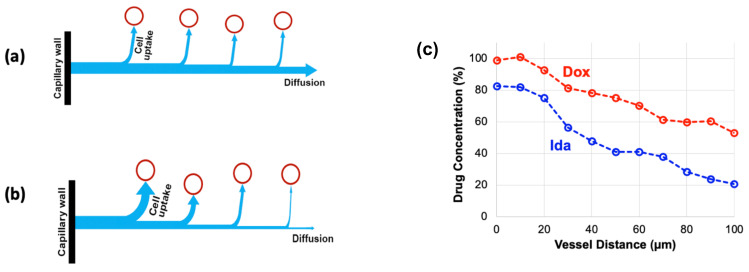
Rapid cell uptake produces steep radial drug concentration gradient. A competition between radial drug diffusion and cellular uptake dictates the radial drug concentration gradient. (**a**) Schematics indicating transport kinetics for a drug with slow cell uptake (e.g., DOX). Diffusion is dominating over cell uptake, allowing drug penetration distant from the capillary. Blue arrows indicate drug transport, red circles represent cancer cells. (**b**) For a drug with rapid cell uptake (e.g., ACLA, IDA), the uptake by cells close to the capillary depletes drug available for more distant cells. (**c**) A prior in vivo study demonstrated a steeper radial concentration gradient for IDA (rapid uptake) compared to DOX (slower uptake), shown after 60 min hyperthermia [105]. Figures reproduced from [172] (published under CC BY license).

Interestingly, the drugs with rapid cell uptake also produced a steep radial concentration gradient surrounding the capillaries (Figure 12c–e). This is because drug uptake by cells close to the capillaries depletes drug amount available for cell uptake further distant from the vessels. For drugs with slower uptake, radial diffusion dominates, resulting in a more uniform radial concentration gradient (Figure 13a). For drugs with rapid cell uptake, cellular uptake dominates, resulting in a steeper gradient (Figure 13b). The radial concentration gradient thus depends on the competition between cellular uptake and radial drug diffusion. This more pronounced gradient for drugs with rapid cell uptake has been also demonstrated in the earlier mentioned prior in vivo study for idarubicin (rapid cell uptake) and doxorubicin (slow cell uptake) (Figure 13c) [105].

## 4. Impact of Hyperthermia Method on Drug Delivery

Various hyperthermia methods and devices have been used in clinical and preclinical studies in combination with TSL. Each heating device induces a different temperature profile in tissue, has varying penetration depth and different heating dynamics. Thus, the choice of the hyperthermia device will have substantial impact on the drug delivery from TSL. Depending on anatomic location and size of the targeted tumor(s), the hyperthermia device needs to be carefully selected and heat delivery optimized.

### 4.1. Temperature

Ideally, the target tissue volume (e.g., tumor) is uniformly exposed to temperatures within the optimal range of ideal drug release from TSL. For the more recent TSL formulation, that corresponds to minimum tissue temperatures of ~40 °C. In addition, maximum temperatures should be limited to avoid reduced blood perfusion. Reduced perfusion takes place above ~43–45 °C in animal tumors [124]. Thus, ideally the target tissue volume is heated to a narrow temperature range of 40–42 °C [173]. While this may be feasible for small rodent tumors, for large human tumors such uniform heating is technically quite challenging [14]. 

In addition to temperature of the target tissue, body temperature can have a significant impact on drug delivery with TSL. Plasma stability is in part due to drug leakage from TSL in systemic plasma at body temperature. A slightly increased body temperature, even if still within the physiological temperature range of the animal or human (e.g., 38–39 °C), may result in premature drug leakage within systemic circulation [30,149]. This can substantially reduce drug delivery as less drug is available for release. Adequate monitoring and control of core body temperature is therefore important.

### 4.2. Hyperthermia Duration and Timing

**Figure 14:**
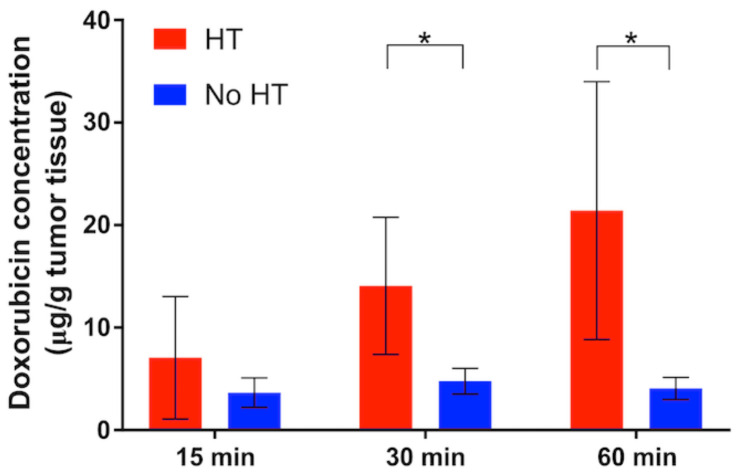
Extended hyperthermia duration increases drug uptake. Tumor drug concentration (doxorubicin) was quantified for tumors receiving hyperthermia for 15, 30 or 60 min (red bars), and for contralateral tumors with no hyperthermia (blue bars) in mice. A regression analysis identified hyperthermia duration as significant predictor of tumor drug uptake (*p* = 0.02). * indicates significance (*p* < 0.05). Figure reproduced from [30] (published under CC BY license).

The hyperthermia duration used in past studies with TSL varies widely, between 2–60 min [30,161]. Multiple more recent studies have demonstrated that longer hyperthermia duration enhances tumor drug uptake (Figure 14) [30,161,162,174,175]. We described earlier that the AUC of the plasma concentration calculated during hyperthermia correlates with the amount of drug released during heating, and therefore predicts tumor drug uptake (Figure 9; Equation (S5)) [149,161,162,163]. This also explains why longer hyperthermia duration enhances drug delivery, since a longer heating duration results in a larger AUC. At some time point, there will however be limited additional benefit of extending the heating duration—i.e., when plasma concentration of TSL-encapsulated drug has decreased substantially.

In addition to hyperthermia duration, the timing of hyperthermia in relation to the administration of TSL-encapsulated drug impacts drug delivery. A past study showed that timing of hyperthermia while keeping duration constant impacts drug delivery [161]. Another recent study demonstrated in vivo that the optimization of hyperthermia timing relative to TSL administration as to maximize plasma AUC also maximizes tissue drug delivery [163].

**Figure 15:**
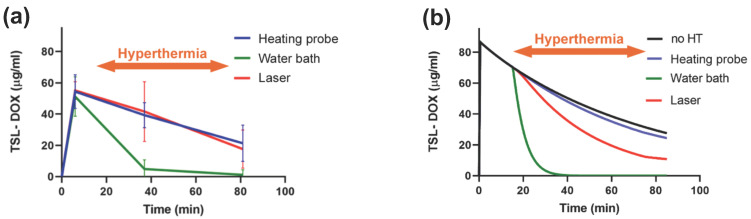
Volumetric heating rapidly depletes systemically available TSL-encapsulated drug. (**a**) Plasma pharmacokinetics of mice injected with TSL-Dox is shown, for three heating modalities. The focal heating modalities (heating probe, laser) have little impact on plasma concentration (e.g., plasma half-life was similar to a prior study in the same animal model without heating [30]). In contrast, water bath heating—where the whole limb is exposed to heat rather than just the tumor—rapidly depletes encapsulated drug in systemic plasma. (**b**) Pharmacokinetic model shows similar results at higher temporal resolution, and includes results in unheated mice (‘no HT’). Figures reproduced from [162] (published under CC BY license).

In many cases, substantial amounts of encapsulated drug will remain in systemic circulation after completion of hyperthermia (Figure 9a, Figure 15). Since no further tumor delivery occurs after completion of heating (Figure 2b) [22,30], this systemically remaining drug does not contribute therapeutically. A recent study presented an approach that removes this remaining encapsulated drug from systemic circulation to reduce systemic toxicities [148].

### 4.3. Volume of Hyperthermia

The hyperthermia volume (volume where significant drug release occurs, e.g., total volume of tissue heated to >40 °C) varies between hyperthermia methods. A recent study demonstrated that the hyperthermia volume can have a substantial impact on the amount of drug delivered to tumors [162]. When a large tissue volume relative to the total body volume is exposed to hyperthermia, large amounts of TSL-encapsulated drug are released. As result, the available encapsulated drug in systemic circulation can be depleted quite rapidly (Figure 15). In such cases, extending the hyperthermia duration beyond the time when all the encapsulated drug has been released does not enhance tumor drug uptake. For example, one prior study showed no difference in tumor uptake between 15 min and 60 min water bath hyperthermia [162].

Two animal studies where water bath heating was employed to heat the entire tumor bearing leg demonstrated significantly lower drug delivery to tumors compared to focal hyperthermia methods such as infrared lasers [162,176]. In both studies, the more focused infrared laser hyperthermia resulted in 2–3 times higher tumor drug uptake compared to volumetric hyperthermia from a water bath. Notably, water bath hyperthermia has been very widely used in TSL studies with rodents [7,8,21,112,128,151,172,176,177,178], and tumor uptake may have been suboptimal in some of these prior studies due to large volumetric heating. The plasma level of TSL-encapsulated drug after hyperthermia completion compared to unheated control animals provides information on how much encapsulated drug has been released during heating (Figure 15b).

While the described mechanism has only been established more recently, there are multiple earlier studies that reported a substantially reduced plasma concentration of TSL-encapsulated drug following hyperthermia, compared to unheated control animals [6,105,112,151,155,176]. In all these studies, it is likely that the amount of drug delivered to tumors was reduced due to large-volume hyperthermia. By how much the delivery was reduced could be estimated by comparing the plasma AUC during heating between animals with and without hyperthermia (Figure 9 and Figure 15).

These observations also have clinical relevance, since various HT devices have been employed in combination with TSL in human patients [129,131,132,133,135,173,179,180]. Additionally, in some cases—such as for the treatment soft tissue sarcoma or for chest wall recurrences after breast cancer—large tissue volumes may be exposed to HT. This could rapidly deplete TSL-encapsulated drug, similarly to the discussed preclinical studies.

### 4.4. Review of Available Hyperthermia Devices

Below, we briefly review hyperthermia devices that have been used in combination with TSL. Table 3 provides an overview of devices. The various hyperthermia devices for human and preclinical application have been reviewed in greater detail in prior publications [181,182,183].

#### 4.4.1. Hyperthermia Devices for Human Use

All clinical studies in humans listed below used the same commercial formulation of thermosensitive liposomal doxorubicin (TSL-Dox), ThermoDox® [135].

Tumor ablation is a clinically used cancer therapy where the cancerous tumor is directly killed by heat, generally at temperatures above 50 °C. In clinical trials, patients with primary liver cancer have been treated with a combination of tumor ablation (specifically, radiofrequency ablation) and TSL-Dox [129,135,173,179,180]. The rationale for this combination is to kill central tumor regions by heat alone and release a therapeutic chemotherapy dose in the margin where temperatures are not adequate for complete cell kill [135,147,161]. Unfortunately, past Phase III trials combining radiofrequency ablation and TSL-Dox in humans with liver cancer have been unsuccessful [173,180]. There are multiple possible reasons for these failures, including selection of patients and drug selection, among others [173,184].

For recurrent chest wall cancer in human patients, microwave hyperthermia has been employed to expose large skin regions and underlying tissue to hyperthermic temperatures [131].

Finally, high-intensity focused ultrasound (HIFU) has been employed in Phase I trials: for targeted drug delivery to patients with liver tumors [133]; in solid pediatric tumors [132]; and in an upcoming trial for pancreatic cancer [173]. HIFU employs ultrasound focused non-invasively into deep tissue regions and enables spatial targeting with millimeter accuracy. Often, HIFU is guided by MR thermometry, enabling real-time monitoring of tissue temperature and accurate temperature control [185].

#### 4.4.2. Hyperthermia Devices for Animal Use

Below, we briefly review those devices that have been used in animal studies in combination with TSL. Two trials in privately owned companion animals—one in dogs and one in cats—used microwave hyperthermia devices. The feline trial employed a microwave applicator for human use to treat sarcomas with a DPPG2-based TSL-Dox formulation [155]. The canine trial used a proprietary microwave hyperthermia applicator to treat sarcoma and carcinoma with the commercial TSL-Dox formulation ThermoDox® [186].

Some studies used tumor ablation devices for humans, either without modification in porcine studies [147,161], or adapted for canine and mouse models [149,187]. One study in normal porcine bladder used irrigation of warm water inside the bladder [188].

Likely the most widely used heating method in rodents—in part due to its simplicity—is the water bath, where the limb including tumor is immersed in heated water [7,8,21,112,128,151,172,176,177,178]. As noted above, while heating is very uniform, a disadvantage of water bath hyperthermia is that a comparably large tissue volume is exposed to heat that likely results in rapid depletion of the available TSL-encapsulated drug in systemic circulation [162].

**Figure 16:**
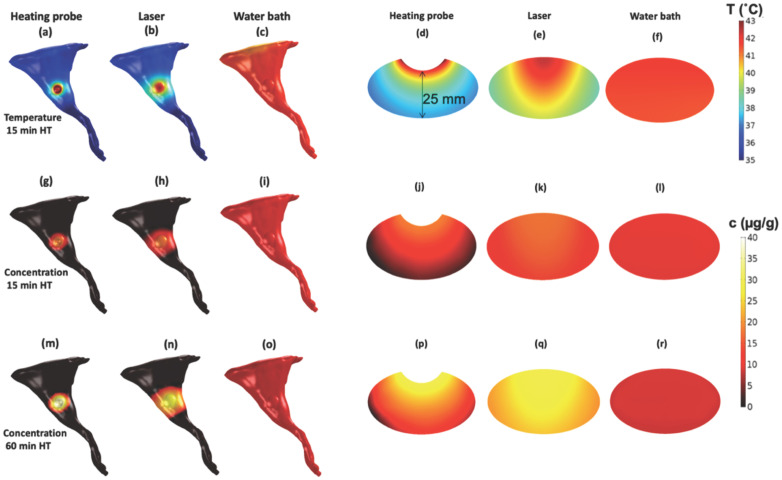
Effect of hyperthermia device on drug delivery. A prior study presented 3-D computer model results on three hyperthermia (HT) devices, assuming a subcutaneous tumor on a mouse hindlimb. The temperature profile after 15 min HT (=steady state profile) is shown for a heating probe, an infrared laser, and water bath heating on the hindlimb surface (**a**–**c**), and in a tumor cross section (**d**–**f**). The resulting drug concentration is shown after 15 min HT for the three devices, again on the limb surface (**g**–**i**), and in a tumor cross section (**j**–**l**). The resulting drug concentration is shown after 60 min HT on the limb surface (**m**–**o**), and in a tumor cross section (**p**–**r**). Figures reproduced from [162] (published under CC BY 4.0 license).

A few studies used focused light sources to induce localized hyperthermia such as infrared lasers [113,114,162,176,189] and cold light lamps [176,190]. Such light sources are attractive for heating subcutaneous tumors since they can be easily targeted and provide often adequate heat penetration for small rodent tumors (Figure 16e).

HIFU has been widely used in combination with TSL in both large and small animal models [17,141,142,143,145,146,163,174,175,191,192,193,194,195,196,197,198,199,200,201,202,203,204]. Similar to human studies with HIFU, it is often combined with MR thermometry to provide non-invasive monitoring and control of temperature.

Intravital microscopy has been used in several studies to monitor drug delivery from TSL at the microscopic level, where custom-designed heating systems based on heating elements, radiofrequency hyperthermia, or microwave hyperthermia have been employed [205].

Finally, a few studies have used custom-designed heating probes applied directly to tumors [30,162,206], though such applicators provide very limited heat penetration (Figure 16d).

The type of device used and resulting temperature distribution significantly impact drug delivery from TSL, as visualized in a recent computer modeling study (Figure 16) [162].

**Table 3:** Heating devices used in human and animal studies with TSL.

	Heating Device	Tem-Perature	Target Tissue	Heating Duration	Device Advantages	Device Limitations	Refs.
Clinical human trials	Radio-frequency ablation	Up to ~100 °C	Primary liver tumors (Phase III trial)	Variable (multiple sequential applications)	Central tumor kill by cytotoxic temperatures >50 °C	Drug delivery limited to margin of heating zone (~40–45 °C)	[129,135,173,179,180]
	Microwave hyperthermia	40.0–42.0 °C	Recurrent chest wall breast cancer (Phase I trial)	60 min	Hyperthermia of large tissue volume		[131]
	High-intensity focused ultrasound (HIFU)	42 °C	Primary liver tumors (Phase I trial); Pancreatic cancer; Pediatric solid tumors (Phase I trial)	30 min	Non-invasive heating of deep tissue regions; excellent spatial targeting	HIFU cannot penetrate air or bone; thermometry is technically complex, and/or expensive (MR thermometry)	[132,133,173]
Animal studies	Radio-frequency ablation	Up to ~100 °C	Normal porcine liver; mouse tumors	5, 12 min and 30 min (porcine liver; 3 min (mouse tumors)	Central tumor kill by cytotoxic temperatures >50 °C	Drug delivery limited to margin of heating zone (~40–45 °C)	[147,161,187]
	Water bath	40–43 °C	Subcutaneous tumors	60 min	Simplicity; Uniform heating	Large heating volume (see [162])	[7,8,21,112,128,151,172,176,177,178]
	Laser (Red or Near-Infrared (760–1000 nm))	40–43 °C	Subcutaneous tumors	15–60 min	Non-contact; spatially targeted	Penetration depth limited to ~1–2 cm	[113,114,162,176,189]
	High intensity focused ultrasound (HIFU)	40–43 °C	Subcutaneous tumor	2–40 min	Non-invasive heating of deep tissue regions; excellent spatial targeting	HIFU cannot penetrate air or bone; Most studies use MR thermometry (expensive)	[17,141,142,143,145,146,163,174,175,191,192,193,194,195,196,197,198,199,200,201,202,203]
	Microwave hyperthermia	40–44 °C	Sarcomas (feline, canine); carcinomas (canine); subcutaneous rat tumors	90 min (canine); 60 min (feline); 15 min (rat tumors)	Microwave antenna with directional heating (rat tumors)		[148,173]
	Custom heating probes	45 °C at probe surface	Subcutaneous tumors	30–60 min		Heating penetration limited	[30,206]

## 5. Impact of Tumor Properties

### 5.1. Tumor Perfusion and Transit Time

We described earlier that tissue transit time is of primary relevance for drug delivery with TSL based on the intravascular triggered release paradigm. Tissue transit time describes the time that plasma spends within the vasculature of the target tissue segment (note that red blood cells typically spend longer than plasma within the same vasculature due to interaction with the small-diameter capillaries [115]). This transit time is the duration available for TSL to release the encapsulated drug (Figure 4 and Figure 5). We discussed earlier how TSL release time impacts delivery (Figure 10); in this prior figure, a certain tumor tissue transit time (=5 s) was assumed. However, it is not just the TSL release time that is relevant; to be more exact, it is the ratio between TSL release time and transit time that is relevant. This ratio determines the amount of drug released while TSL pass through the capillaries of the target tissue (Supplementary Material File S1, Equation (S2)) [20]. For example, delivery to tumors with short transit times of ~2 s will therefore be less efficient compared to tumors with longer transit times.

Tumor perfusion describes the volume of blood or plasma passing through tissue (e.g., mL plasma per g tissue per minute). Tumor perfusion and transit time are inversely related [20], i.e., a higher perfusion is typically associated with a shorter transit time. In addition, perfusion and transit time vary spatially within tissue (e.g., tumors). This spatial heterogeneity will therefore contribute to heterogenous drug delivery within tumors.

### 5.2. Tumor Microenvironment

The tumor microenvironment includes cancer cells, stromal cells, immune cells, blood vessels and the extracellular matrix. The tumor microenvironment is highly heterogenous, and here we will focus on microenvironmental aspects that affect TSL delivery. The extracellular matrix (ECM) (i.e., dense stroma) is known to represent a barrier for drug delivery [14,207], and a prior study identified high collagen content (a major component of the ECM) as contributing to poor treatment response following TSL-based delivery [114]. This prior study also identified a second factor of the microenvironment as contributing factor to poor response: hypoxia. Hypoxia is the result of low vascular density. The low vascular density results in large distances between neighboring capillaries, with limited penetration of oxygen into regions distant from any capillary. Hypoxia is generally assumed as contributing towards poor tumor response to drug-based therapies [207,208,209]. In addition, drug penetration into hypoxic regions is lower, since the large distance from neighboring capillaries limits drug penetration in addition to oxygen penetration [209] (see also Figure 12).

In addition, several biophysical parameters dependent on the tumor microenvironment impact drug delivery. These include for example the vascular fraction, extravascular fraction, and vascular permeability [20,207]. The latter depends both on the drug and on vascular biology, and determines in part the drug extraction fraction (EF) discussed earlier.

Finally, the microenvironment also indirectly affects transit time and perfusion of tumors, e.g., due to variations in vascular geometry and vessel density.

### 5.3. Cancer Cell Properties

The varying therapeutic response of different tumor types is further affected by properties of the cancer cells. It is we well known that different cancer cell types have varying sensitivity to a particular drug [111,172,210]. Further, we described earlier that cell uptake kinetics varies as well between cancer cell types [171,172,211]. This varying uptake kinetics impacts delivery to cancer cells, and may impact efficacy of therapy as well. In addition, proliferative properties of cancer cells can affect treatment response. A prior study with five tumor types analyzed in vitro cancer cell properties and in vivo tumor response after treatment with TSL-Dox. The in vitro doubling time was identified as significantly correlating with in vivo tumor response (i.e., tumor growth time) [111].

## 6. Other Hyperthermia Effects

Hyperthermia induces a variety of biological effects that impact both drug transport and cytotoxicity, and therefore hyperthermia also indirectly impacts delivery and efficacy of TSL-based therapies. Hyperthermia has been widely used as cancer therapy in human clinical trials with limited side effects, usually in combination with chemo- or radiation-therapy [212,213]. Relevant for TSL-encapsulated chemotherapeutics, hyperthermia is a well-known chemosensitizer that enhances the efficacy of many chemotherapy agents. In part, this is due to enhanced cellular uptake of drugs at elevated temperatures [214]. In addition, hyperthermia inhibits multiple DNA repair mechanisms, providing a synergistic cytotoxicity with agents that cause DNA damage [215]. In addition, hyperthermia transiently enhances vascular permeability which can improve transvascular transport of macromolecules [216], or can enable drug transport across the blood–brain barrier [149]. Furthermore, hyperthermia improves tumor perfusion, which is particularly helpful in poorly perfused, often hypoxic tumor regions. There, re-oxygenation can improve therapy response, while enhanced perfusion can improve drug delivery [214]. Finally, hyperthermia locally stimulates the immune system, which can improve anti-tumor response [214,217]. Most of these effects depend on both temperature and heating duration, and thus may require additional consideration when devising heating algorithms to take advantage of these effects while also providing optimal TSL drug delivery.

## 7. Recommendations for Preclinical TSL Studies

Below, we provide some guidelines based on our review of preclinical TSL studies in rodents that may be helpful for future studies:

- Measure in vitro TSL release kinetics at early time points, with the first measurement ideally within 5 s or less. In addition, a physiologically relevant buffer should be used (e.g., plasma or serum), since the buffer affects drug release (Figure 6d) [121].
- Initiate hyperthermia (HT) either before bolus administration of TSL, or as soon as practical after administration. This is to maximize the plasma-AUC, which correlates with tumor drug uptake (Figure 9) [149,161,162,163]. Pre-heating is particularly advantageous in cases when heating of the tumor requires some time (depending on heating method).
- Use a heating method that ensures heating of the whole tumor while avoiding extensive exposure of normal tissues. To ensure adequate tumor heating for subcutaneous tumors, at minimum, temperature at the distal edge of a subcutaneous tumor should be measured to confirm that the whole tumor is exposed to hyperthermic temperatures where the employed TSL have optimal release (~40–43 °C in most cases). While MR thermometry or ultrasound thermometry are often not available, such methods would be ideal to ensure targeted tumor heating. As discussed above, water bath hyperthermia is not ideal for rodent studies and can result in reduced delivery [162].
- Obtain a blood sample after completion of HT, to quantify drug concentration and ensure that available encapsulated drug has not been depleted. A comparison to a non-heated control group confirms if any depletion is due to HT, rather than from systemic TSL elimination/leakage. An additional blood sample following TSL administration and before HT would be valuable (e.g., for estimating the plasma-AUC as in Figure 9). While the required HT duration for therapeutic effect depends on many factors such as drug, tumor model, etc., in general, extending the HT duration enhances tumor drug uptake assuming that TSL-encapsulated drug is still in circulation.
- Provide optimal thermal support and monitor the core temperature of animals during studies. Due to anesthesia, rodents are not able to regulate their core temperature and require thermal support. However, extensive thermal support may elevate core temperature above normal. Prior studies have shown that elevated core temperature (>37 °C) resulted in premature drug leakage from TSL, even though thermal support was at 37 °C [30]. Conversely, a reduced core temperature will make it more difficult to raise tumor temperature to ranges required for release. Thus, ideally the core temperature should be continuously monitored and regulated to ~36–37 °C by adjusting thermal support as necessary.

## 8. Conclusions

**Figure 17:**
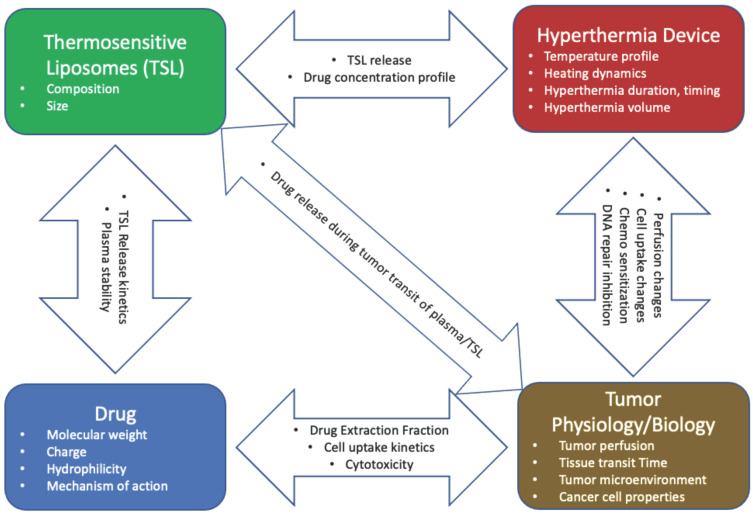
Interactions between TSL, drug, hyperthermia device, and tumor physiology/biology. The properties listed inside the arrows result from interactions between specific components (e.g., TSL and drug). These interactions must be carefully considered when designing TSL–drug-device combinations for optimal delivery.

Drug delivery by TSL depends on complex interactions between the liposomes, the drug, the hyperthermia device, and tumor physiology/biology (Figure 17). Optimal delivery by TSL requires rapid drug release from liposomes, ideally combined with a drug that is quickly taken up by tissue and cancer cells. In addition, selection of an adequate hyperthermia device that can expose the target tissue to temperatures of ~40–42 °C with limited exposure of non-targeted tissues is of importance. Many of the discussed concepts are applicable to other heat-activated nanoparticles, and triggered drug delivery systems in general.

## Figures and Tables

**Table 1:** *Fast-release TSL formulations.* Release times (see Figure 7) were estimated if possible, or an upper limit was provided (e.g., <20 s); in the latter cases, release at the first measured time point is shown in brackets. Buffer used for release measurement is indicated, since buffer affects release kinetics [121].

TSL Composition (Molar Ratio)	Drug	Release Time [Temp.]	Buffer	In Vivo Plasma Half-Life *(Species)*	Refs.
DPPC:MSPC:DSPE-PEG2000 (86:10:4)	Doxorubicin	3 s [40 °C]	human plasma	0.96 h *(human)*; 1–2 h *(rabbit)*; 4.8 h *(pig)*	[127,128,129,140,145,146,147]
DPPC:MSPC:DSPE-PEG2000 (85.3:9.7:5)	Doxorubicin	4 s [41 °C]	PBS	0.93 h *(mouse)*; 0.96 h *(rat)*; 0.75 h *(dog)*	[30,148,149]
DPPC:DSPC:DSPE-PEG2000 (70:25:5)	Doxorubicin	~5–10 s [42 °C]	FBS	>1 h *(mouse)*	[105]
DPPC:DSPE-PEG2000:Ch:mELP	Doxorubicin	<5 s [41–42 °C]	FBS + culture media	2.0 h *(mouse)*	[150]
DPPC:DSPC:DPPG1 (50:20:30)	Doxorubicin	<20 s [42 °C] (92.2% release @ 20 s)	HEPES buffered saline	1.4 h *(rat)*	[151]
DPPC:DSPC:DPPG2 (50:20:30)	Doxorubicin	<20 s [42 °C] (~75% release @ 20 s)	HEPES buffered saline	~1 h *(pig)*; 1.6–2.4 h *(rat)*; 0.4–0.7 h *(cat)*	[151,152,153,154,155]
EYPC:Chol:Peg-PE:poly(EOEOVE-OD4) (50:45:4:2)	Doxorubicin	~1 min [43 °C]	HEPES buffered saline	-	[156]
DPPC:Brij78	Doxorubicin	~1 min [42 °C]	FBS	0.5 h *(mouse)*	[157]
DOPE:EPC:chol-pHPMAlac (70:25:5)	Doxorubicin	~2 min [42 °C]	HEPES buffered saline	-	[158]
DPPC:DSPC:DSPE-PEG2000 (60:35:5)	Idarubicin	<1 s [42 °C]	FBS	>1 h *(mouse)*	[105]
DPPC:DSPC:DSPE-PEG2000 (80:15:5)	Gemcitabine	<2 min [42 °C] (90% release @ 2 min)	FBS	~2 h *(mouse)*	[143]
DPPC:MSPC:DSPE-PEG2000 (86:10:4)	Gemcitabine	~30–60 s	FBS:saline (1:1)	-	[34]
DPPC:Brij78	Gemcitabine	~30–60 s	FBS:saline (1:1)	~2 h *(mouse)*	[34]
DPPC:Brij78	Oxiplatin	~30–60 s	FBS:saline (1:1)	~1 h *(mouse)*	[34]
DPPC:DSPC (90:10)	Cisplatin	3–5 s [43 °C]	rat plasma	~1 h *(mouse)*	[125,126,159]
DPPC:DPPG:MSPC:DSPE-PEG2000 (57.7:28.9:9.6:3.8)	Cisplatin	<5 min [42 °C] (90% release @ 5 min)	0.9% saline	~1.5 h *(mouse)*	[113]
DPPC:MSPC:DSPG:DSPE-PEG2000 (82:8:10:4)	Epirubicin	~4 min [41–43 °C]	PBS	0.2 h *(rat)*	[160]
DPPC:MSPC:DSPE-PEG2000 (86:10:4)	Alvespimycin	<30 s [42 °C] (90% release @ 30 s)	BSA in PBS	0.2 h *(mouse)*	[80]

DPPC: 1,2-Dipalmitoyl-sn-glycero-3-phosphocholine; DSPC: 1,2-distearoyl-sn-glycero-3-phosphocholine; MSPC: 1-stearoyl-2-hydroxy-sn-glycero-3-phosphatidylcholine; DPPG: 1,2-dipalmitoyl- sn-glycero-3-phosphoglycerol; PE: poly ethylene; PEG: polyethylene glycol; Ch: Cholesterol; EYPC: egg yolk phosphatidylcholine; EOEOVE: 2-(2-ethoxy)ethoxyethyl vinyl ether; mELP: modified elastin-like polypeptide; Brij78: proprietary surfactant (main component: eicosaethylene glycol octadecyl ether); pHPMAlac: 2-Hydroxypropyl methacrylamide mono/dilactate polymers; PBS: phosphate buffered saline; FBS: fetal bovine serum; BSA: bovine serum albumin.

**Table 2:** Extraction fraction (EF) of chemotherapy agents that have been encapsulated in TSL.

Drug	Extraction Fraction (EF)	Source
Idarubicin	~1	[167]
Gemcitabine	0.55–0.89	[168]
Oxaliplatin	0.47	[169]
Doxorubicin	0.45–0.5	[166]
Cisplatin	0.24	[170]

## Supplementary Materials

The following are available online at https://www.mdpi.com/article/10.3390/cancers15020398/s1, Supplementary Material File S1: Supplementary information.
