# Supplementary material for: Review of the Delivery Kinetics of Thermosensitive Liposomes
Source: Cancers (Basel). 2023 Jan 7;15(2):398. doi: 10.3390/cancers15020398 (PMC9856714; doi:10.3390/cancers15020398)
Supplement: Supplementary file 1 [file cancers-15-00398-s001.zip › cancers-2050893-supplementary.pdf]

Supplementary Information

S1. Plasma AUC correlates with total drug released

Below we show that the area-under-the-curve (AUC) of TSL-encpasulated drug concentration in systemic plasma, calculated during hyperthermia, correlates with total amount of drug released. In the following equations, the units are listed in square brackets.
The amount of encapsulated (liposomal) drug entering the tumor (μg drug per mL tumor per second) is:

$$\acute{Q}_{Lip}{}^{T}\,[\frac{\mu g}{sec}] = c_{p\_Lip}{}^{S} \cdot F_p{}^{T} \cdot V^{T} \quad \text{(Equation S1)}$$

We assume TSL release can be approximated by a linear function (Figure 7). Then, the fraction of encapsulated drug released (*R*) while TSL pass through the tumor capillaries depends on release time t_{rel} and tissue transit time (*TT*):

$$R = \frac{TT}{t_{rel}} \quad ; [t_{rel} > TT]$$

$$R = 1 \quad ; [t_{rel} < TT] \qquad \text{(Equation S2)}$$

E.g., if the TSL release time is twice as long as the transit time, 50% of the encapsulated drug would be released.

The amount of drug released within the tumor (μg drug per second) is then:

$$\acute{Q}_{Drug}{}^{T}\,[\frac{\mu g}{sec}] = R \cdot c_{p\_Lip}{}^{S} \cdot F_p{}^{T} \cdot V^{T} \qquad \text{(Equation S3)}$$

Assuming a hyperthermia duration t_{heat}, then the total amount of drug released within the tumor (μg drug) is:

$$Q_{Drug}{}^{T}[\mu g] = R \cdot c_{p\_Lip}{}^{S} \cdot t_{heat} \cdot F_p{}^{T} \cdot V^{T} \qquad \text{(Equation S4)}$$

Until now, we assumed that the systemic plasma concentration of liposomal drug ($c_{p\_Lip}{}^{S}$) is constant. If we now assume that this concentration varies with time ($c_{p\_Lip}{}^{S}(t)$), then the term '$c_{p\_Lip}{}^{S} \cdot t_{heat}$' in Equation S4 becomes an integral. If we assume heating starts at time t_0, obtain:

$$Q_{Drug}{}^{T}[\mu g] = F_p{}^{T} \cdot V^{T} \cdot R \cdot \int_{t_0}^{t_0+t_{heat}} c_{p_{Lip}}{}^{S}(t)\,dt \quad \text{(Equation S5)}$$

The integral in equation S5 is the area-under-the-curve (AUC) of the systemic plasma concentration of TSL-encapsulated drug, calculated during hyperthermia. I.e., the total amount of drug released in the tumor during hyperthermia ($Q_{Drug}{}^{T}$) correlates with this AUC. In addition, the amount of drug released depends on *R*, which depends on the TSL formulation and temperature.

Table 1. List of variables and parameters used in Equation S1-S5.

Variable/Parameter [Units]	Description
$c_{p\_Lip}{}^{S}$ [μg/mL]	TSL-encpasulated drug concentration in systemic plasma
V^{T} [mL]	Tumor volume
t_{rel} [sec]	TSL release time (see Figure 7)
TT [sec]	Tumor transit time (time required for plasma to pass through tumor capillaries, see Figure 4)
R []	Fraction of TSL-encapsulated drug released
$Q'_{Drug}{}^{T}$ [μg/sec]	Amount of drug released in tumor per second
$Q'_{Drug}{}^{T}$ [μg]	Total amount of drug released in tumor during hyperthermia
t_{heat} [sec]	Hyperthermia duration
t_0 [sec]	Time when hyperthermia starts

S2. Reviewed nanoparticle publications

For comparison between TSL and other nanoparticle formulations in terms of tumor uptake (Figure 3), we used a prior review that compared 117 studies on nanoparticle formulations between 2005-2015 [1], and added TSL studies within that same time period. In addition, we performed a literature search between 2016-2022 to include newer nanoparticles as well in our comparison, using the same search criteria and normalization methods as in Wilhelm et al. [1]. Specifically, Using GoogleScholar and SciFinder search engines, combinations of keywords such as nanoparticles, biodistribution, quantitative, %ID, and thermosensitive liposomes were searched in English-language, peer-reviewed journals between 2016-2022. Studies not directly reporting quantitative information such as tumor drug uptake as percent injected dose within specific time periods (%ID/g) or where the tumor AUC could not be calculated by reported details (e.g., injected dose, μg drug per g tumor; for volumes, 1 mL tumor was considered as 1.2g) were not included. Total tumor AUCs for concentrations (C, %ID/g) and time (t, in hours) were calculated by the linear trapezoid method:

$$AUC = \sum_{i=1}^{n}(0.5\,(C_i + C_{i-1}) \cdot (t_i - t_{i-1})) \qquad \text{(Equation S6)}$$

Total %ID/g was then normalized by dividing the AUC by the study's reported period of biodistribution measurements in hours. Table 2 lists the prior studies that were considered in addition to the 117 prior studies reviewed by Wilhelm et al [1].

Table 2. Prior studies with passive, active and triggered nanoparticles between 2016-2022 were reviewed, as well as TSL studies. Tumor uptake was normalized as described by Wilhelm et al [1]. Studies where 'Type' is succeeded by an asterisk (*) indicate that tumor drug uptake was measured only at a single time point. Typically, the tumor uptake AUC (Equation S6) underestimated true AUC in these studies since tumor concentration is assumed zero immediately after the measured time point.

Year	Material	Type	Tumor uptake [%ID/g]	Drug	Ref
2007	liposomes	TSL*	12.8	doxorubicin	[2]
2010	liposomes	TSL*	4.5	doxorubicin	[3]
2010	liposomes	TSL*	9.6	doxorubicin	[3]
2010	liposomes	TSL*	10	doxorubicin	[3]
2010	liposomes	TSL*	14.8	doxorubicin	[3]
2010	liposomes	TSL*	19	doxorubicin	[3]
2011	liposomes	TSL*	15	doxorubicin	[4]
2011	liposomes	TSL*	6.6	doxorubicin	[4]
2013	liposomes	TSL*	37.5	gemcitabine	[5]
2014	liposomes	TSL*	0.9	cisplatin	[6]
2016	gold hybrid	triggered	7.5	doxorubicin	[7]
2016	polymer (iron-PEG)	active	6	doxorubicin	[7]
2016	hybrid (bismuth-PDA)	triggered	4.6	doxorubicin	[8]
2016	lipid-polymer (PLGA)	active	1.1	doxorubicin	[9]
2016	polymer (PLGA)	passive	2.6	emodin	[10]
2016	polymer (HPMA)	passive	7.9	doxorubicin	[11]
2016	polymer (heparin-deoxycholate)	passive	1.2	doxorubicin	[12]

2016	polymer (PEG-PDTC)	active	2.6	doxorubicin	[13]
2016	lipid-polymer hybrid	passive	0.8	doxorubicin	[14]
2016	polymer (inulin)	active	5.0	epirubicin	[15]
2016	polymer (PCSSL)	triggered	3.6	doxorubicin	[16]
2016	polymer (PEG)	passive	0.9	paclitaxel	[17]
2016	lipid-polymer (zinc)	passive	4.6	cisplatin, siRNA	[18]
2016	polymer (chitosan)	active	1.3	siRNA	[19]
2016	folate-cobalt	active	1.3	doxorubicin	[20]
2017	liposomes	TSL*	0.8	cisplatin	[21]
2017	iron oxide	passive	6.6	enzymes	[22]
2017	silica-lipid	triggered	11.2	doxorubicin	[23]
2017	copper hybrid	active	8.9	artesunate	[24]
2017	polymer (chitosan)	triggered	3.5	doxorubicin	[25]
2017	silica	passive	1.0	doxorubicin, miRNA	[26]
2017	silica-liposome	passive	6.1	doxorubicin, cytokine	[27]
2017	polymer (PLys-PGlu-PEG-PCL)	passive	1.3	cabazitaxel	[28]
2017	polymer (PLGA)	passive	0.47	garcinol	[29]
2017	polymer (POEA)	triggered	11.4	doxorubicin	[30]
2017	polymer (PEG-CM cellulose)	passive	4.2	podophyllotox in	[31]
2017	polymer (PLGA-PEG)	triggered	0.8	paclitaxel	[32]
2017	polymer (heparin)	active	2.6	cisplatin	[33]
2017	polymer (PEG)	triggered	11.2	doxorubicin	[34]
2017	polymer (PLGA-PEG)	passive	3.6	vincristine	[35]
2017	polymer (PEG-TMCC)	passive	1.0	docetaxel	[36]
2017	silica-polydopamine	triggered	14.6	doxorubicin	[37]
2017	hybrid (gold-manganese-PLGA)	triggered	1.2	docetaxel	[38]
2017	polymer (chitosan)	active	2.4	paclitaxel	[39]
2017	polymer (POEA)	triggered	7.6	doxorubicin	[40]
2018	iron oxide-silica	active	13.6	epirubicin	[41]
2018	polymer (PEG-PLGA)	active	3.7	curcumin	[42]
2018	iron oxide-silica	triggered	22.4	doxorubicin	[43]
2018	nanocrystal (albumin)	passive	1.0	paclitaxel	[44]
2018	silica hybrid-copper	triggered	4.6	doxorubicin	[45]

2018	silica	triggered *	2.6	doxorubicin	[46]
2018	protein (albumin)	active	6.1	paclitaxel	[47]
2018	liposomes	active	2.1	doxorubicin	[48]
2018	gold-silica	triggered *	4.3	doxorubicin	[49]
2018	polymer (polyrotaxane)	active	3.75	paclitaxel	[50]
2019	liposomes	TSL*	12.4	doxorubicin	[51]
2019	liposomes	TSL*	11.0	doxorubicin	[52]
2019	polymer (oleyl hyaluronic acid)	passive	1.7	doxorubicin	[53]
2019	hybrid (polymer-liposome)	passive	0.9	oxaliplatin	[54]
2019	Iron acetylacetonate	active*	1.5	*^{18}F	[55]
2019	polymer (PEG-MPE-BzMA)	triggered	4.4	camptothecin	[56]
2019	gold	active*	3.8	cisplatin	[57]
2019	hybrid (lipid-PLGA)	passive*	6	docetaxel	[58]
2019	liposomes	active	11.8	doxorubicin	[59]
2019	hybrid (HMPB-PVP)	triggered	0.8	doxorubicin	[60]
2019	polymer (PLGA)	active*	0.18	doxorubicin	[61]
2020	polymer (PEG)	triggered	1.4	paclitaxel	[62]
2020	polymer (PLGA)	active*	3.9	Epigallo-catechin-3-gallate	[63]
2020	gold	active*	4.5	-	[64]
2020	polymer (PEG-PLA)	passive	2.5	cabazitaxel	[65]
2020	hybrid (gold-polysiloxane)	passive	1.4	radiosensitizer	[66]
2020	polymer (PCD)	passive*	4	enzymes	[67]
2020	liposomes	active*	20	oxaliplatin	[68]
2020	liposomes	active	3.8	radionuclide	[69]
2020	polymer (PDMA)	triggered *	5.6	camptothecin	[70]
2020	polymer (hyaluronan)	triggered *	7.3	7-ethyl-10 hydroxy-camptothecin	[71]
2020	liposomes	triggered *	0.3	In-111 (radionuclide)	[72]
2020	polymer (PAMAM)	triggered	7.9	cisplatin	[73]
2020	gold-silver colloid	passive	1.6	gold-silver	[74]
2021	liposomes	TSL	13.5	idarubicin	[75]
2021	liposomes	TSL	3.5	doxorubicin	[75]

2021	polymer (PAMAM)	triggered	11.6	gefitinib	[76]
2021	polymer (PRES)	passive*	0.85	paclitaxel	[77]
2021	liposomes	active	3.5	radioisotopes	[78]
2021	gold	triggered	16.7	methotrexate	[79]
2021	nanocrystal colloid (vaterite)	passive*	11.0	porphyrazine	[80]
2021	gold	triggered	6.7	doxorubicin	[81]
2022	liposomes	TSL	8.0	doxorubicin	[82]
2022	liposomes	triggered	3.5	doxorubicin	[83]
2022	hybrid (polymer-iron-calcium carbonate)	triggered *	2.25	cisplatin	[84]

References

1. Wilhelm, S.; Tavares, A.J.; Dai, Q.; Ohta, S.; Audet, J.; Dvorak, H.F.; Chan, W.C.W. Analysis of nanoparticle delivery to tumours. *Nature Reviews Materials* **2016**, *1*, 16014.
2. Kong, G.; Anyarambhatla, G.; Petros, W.P.; Braun, R.D.; Colvin, O.M.; Needham, D.; Dewhirst, M.W. Efficacy of liposomes and hyperthermia in a human tumor xenograft model: Importance of triggered drug release. *Cancer Res* **2000**, *60*, 6950-6957.
3. Yarmolenko, P.S.; Zhao, Y.; Landon, C.; Spasojevic, I.; Yuan, F.; Needham, D.; Viglianti, B.L.; Dewhirst, M.W. Comparative effects of thermosensitive doxorubicin-containing liposomes and hyperthermia in human and murine tumours. *Int J Hyperthermia* **2010**, *26*, 485-498.
4. Schmidt, R. Neuartige thermosensitive liposomen zur zielgerichteten therapie solider tumoren. Ludwig-Maximilians-Universität München, 2011.
5. May, J.P.; Ernsting, M.J.; Undzys, E.; Li, S.-D. Thermosensitive liposomes for the delivery of gemcitabine and oxaliplatin to tumors. *Molecular Pharmaceutics* **2013**, *10*, 4499-4508.
6. Dou, Y.N.; Zheng, J.; Foltz, D.W.; R.Weersink; Chaudary, N.; Jaffray, D.A.; Allen, C. Heat-activated thermosensitive liposomal cisplatin (htlc) results in effective growth delay of cervical carcinoma in mice. *Journal of controlled release : official journal of the Controlled Release Society* **2014**, *178*, 69-78.
7. Shi, J.; Chen, Z.; Wang, L.; Wang, B.; Xu, L.; Hou, L.; Zhang, Z. A tumor-specific cleavable nanosystem of peg-modified c60@au hybrid aggregates for radio frequency-controlled release, hyperthermia, photodynamic therapy and x-ray imaging. *Acta Biomaterialia* **2016**, *29*, 282-297.
8. Li, Z.; Hu, Y.; Howard, K.A.; Jiang, T.; Fan, X.; Miao, Z.; Sun, Y.; Besenbacher, F.; Yu, M. Multifunctional bismuth selenide nanocomposites for antitumor thermo-chemotherapy and imaging. *ACS Nano* **2016**, *10*, 984-997.
9. Dai, Y.; Xing, H.; Song, F.; Yang, Y.; Qiu, Z.; Lu, X.; Liu, Q.; Ren, S.; Chen, X.; Li, N. Biotin-conjugated multilayer poly [d,l-lactide-co-glycolide]-lecithin-polyethylene glycol nanoparticles for targeted delivery of doxorubicin. *Journal of Pharmaceutical Sciences* **2016**, *105*, 2949-2958.
10. Liu, H.; Gao, M.; Xu, H.; Guan, X.; Lv, L.; Deng, S.; Zhang, C.; Tian, Y. A promising emodin-loaded poly (lactic-co-glycolic acid)-d-α-tocopheryl polyethylene glycol 1000 succinate nanoparticles for liver cancer therapy. *Pharmaceutical Research* **2016**, *33*, 217-236.
11. Tomalova, B.; Sirova, M.; Rossmann, P.; Pola, R.; Strohalm, J.; Chytil, P.; Cerny, V.; Tomala, J.; Kabesova, M.; Rihova, B., *et al.* The structure-dependent toxicity, pharmacokinetics and anti-tumour activity of hpma copolymer conjugates in the treatment of solid tumours and leukaemia. *Journal of Controlled Release* **2016**, *223*, 1-10.
12. Mei, L.; Liu, Y.; Zhang, H.; Zhang, Z.; Gao, H.; He, Q. Antitumor and antimetastasis activities of heparin-based micelle served as both carrier and drug. *ACS Applied Materials & Interfaces* **2016**, *8*, 9577-9589.
13. Zou, Y.; Fang, Y.; Meng, H.; Meng, F.; Deng, C.; Zhang, J.; Zhong, Z. Self-crosslinkable and intracellularly decrosslinkable biodegradable micellar nanoparticles: A robust, simple and

multifunctional nanoplatform for high-efficiency targeted cancer chemotherapy. *Journal of Controlled Release* **2016**, *244*, 326-335.

14. Zhang, R.X.; Cai, P.; Zhang, T.; Chen, K.; Li, J.; Cheng, J.; Pang, K.S.; Adissu, H.A.; Rauth, A.M.; Wu, X.Y. Polymer–lipid hybrid nanoparticles synchronize pharmacokinetics of co-encapsulated doxorubicin–mitomycin c and enable their spatiotemporal co-delivery and local bioavailability in breast tumor. *Nanomedicine: Nanotechnology, Biology and Medicine* **2016**, *12*, 1279-1290.
15. Zhang, L.; Li, G.; Gao, M.; Liu, X.; Ji, B.; Hua, R.; Zhou, Y.; Yang, Y. Rgd-peptide conjugated inulin-ibuprofen nanoparticles for targeted delivery of epirubicin. *Colloids and Surfaces B: Biointerfaces* **2016**, *144*, 81-89.
16. Huang, P.; Liu, J.; Wang, W.; Zhang, Y.; Zhao, F.; Kong, D.; Liu, J.; Dong, A. Zwitterionic nanoparticles constructed from bioreducible raft–rop double head agent for shell shedding triggered intracellular drug delivery. *Acta Biomaterialia* **2016**, *40*, 263-272.
17. Chen, Y.; Xia, R.; Huang, Y.; Zhao, W.; Li, J.; Zhang, X.; Wang, P.; Venkataramanan, R.; Fan, J.; Xie, W., *et al.* An immunostimulatory dual-functional nanocarrier that improves cancer immunochemotherapy. *Nature Communications* **2016**, *7*, 13443.
18. He, C.; Poon, C.; Chan, C.; Yamada, S.D.; Lin, W. Nanoscale coordination polymers codeliver chemotherapeutics and sirnas to eradicate tumors of cisplatin-resistant ovarian cancer. *J Am Chem Soc* **2016**, *138*, 6010-6019.
19. Nascimento, A.V.; Gattacceca, F.; Singh, A.; Bousbaa, H.; Ferreira, D.; Sarmento, B.; Amiji, M.M. Biodistribution and pharmacokinetics of mad2 sirna-loaded egfr-targeted chitosan nanoparticles in cisplatin sensitive and resistant lung cancer models. *Nanomedicine (Lond)* **2016**, *11*, 767-781.
20. Liu, L.X.; Li, B.X.; Wang, Q.Y.; Dong, Z.P.; Li, H.M.; Jin, Q.M.; Hong, H.; Zhang, J.; Wang, Y. An integrative folate-based metal complex nanotube as a potent antitumor nanomedicine as well as an efficient tumor-targeted drug carrier. *Bioconjugate Chemistry* **2016**, *27*, 2863-2873.
21. Dou, Y.N.; Chaudary, N.; Chang, M.C.; Dunne, M.; Huang, H.; Jaffray, D.A.; Milosevic, M.; Allen, C. Tumor microenvironment determines response to a heat-activated thermosensitive liposome formulation of cisplatin in cervical carcinoma. *Journal of controlled release : official journal of the Controlled Release Society* **2017**, *262*, 182-191.
22. Huo, M.; Wang, L.; Chen, Y.; Shi, J. Tumor-selective catalytic nanomedicine by nanocatalyst delivery. *Nature Communications* **2017**, *8*, 357.
23. Su, J.; Sun, H.; Meng, Q.; Zhang, P.; Yin, Q.; Li, Y. Enhanced blood suspensibility and laser-activated tumor-specific drug release of theranostic mesoporous silica nanoparticles by functionalizing with erythrocyte membranes. *Theranostics* **2017**, *7*, 523-537.
24. Hou, L.; Shan, X.; Hao, L.; Feng, Q.; Zhang, Z. Copper sulfide nanoparticle-based localized drug delivery system as an effective cancer synergistic treatment and theranostic platform. *Acta Biomaterialia* **2017**, *54*, 307-320.
25. Wu, J.; Tang, C.; Yin, C. Co-delivery of doxorubicin and interleukin-2 via chitosan based nanoparticles for enhanced antitumor efficacy. *Acta Biomaterialia* **2017**, *47*, 81-90.
26. Xue, H.; Yu, Z.; Liu, Y.; Yuan, W.; Yang, T.; You, J.; He, X.; Lee, R.J.; Li, L.; Xu, C. Delivery of mir-375 and doxorubicin hydrochloride by lipid-coated hollow mesoporous silica nanoparticles to overcome multiple drug resistance in hepatocellular carcinoma. *International journal of nanomedicine* **2017**, *12*, 5271-5287.

27. Kong, M.; Tang, J.; Qiao, Q.; Wu, T.; Qi, Y.; Tan, S.; Gao, X.; Zhang, Z. Biodegradable hollow mesoporous silica nanoparticles for regulating tumor microenvironment and enhancing antitumor efficiency. *Theranostics* **2017**, *7*, 3276-3292.
28. Gou, J.; Liang, Y.; Miao, L.; Guo, W.; Chao, Y.; He, H.; Zhang, Y.; Yang, J.; Wu, C.; Yin, T., *et al.* Improved tumor tissue penetration and tumor cell uptake achieved by delayed charge reversal nanoparticles. *Acta Biomaterialia* **2017**, *62*, 157-166.
29. Gaonkar, R.H.; Ganguly, S.; Dewanjee, S.; Sinha, S.; Gupta, A.; Ganguly, S.; Chattopadhyay, D.; Chatterjee Debnath, M. Garcinol loaded vitamin e tpgs emulsified plga nanoparticles: Preparation, physicochemical characterization, in vitro and in vivo studies. *Scientific Reports* **2017**, *7*, 530.
30. Yan, G.; Wang, J.; Qin, J.; Hu, L.; Zhang, P.; Wang, X.; Tang, R. Well-defined poly(ortho ester amides) for potential drug carriers: Probing the effect of extra- and intracellular drug release on chemotherapeutic efficacy. *Macromolecular Bioscience* **2017**, *17*, 1600503.
31. Roy, A.; Zhao, Y.; Yang, Y.; Szeitz, A.; Klassen, T.; Li, S.-D. Selective targeting and therapy of metastatic and multidrug resistant tumors using a long circulating podophyllotoxin nanoparticle. *Biomaterials* **2017**, *137*, 11-22.
32. Boissenot, T.; Bordat, A.; Larrat, B.; Varna, M.; Chacun, H.; Paci, A.; Poinsignon, V.; Fattal, E.; Tsapis, N. Ultrasound-induced mild hyperthermia improves the anticancer efficacy of both taxol® and paclitaxel-loaded nanocapsules. *Journal of Controlled Release* **2017**, *264*, 219-227.
33. Wang, J.; Lee, G.Y.; Lu, Q.; Peng, X.; Wu, J.; Wu, S.; Kairdolf, B.A.; Nie, S.; Wang, Y.; Lane, L.A. Quantitative examination of the active targeting effect: The key factor for maximal tumor accumulation and retention of short-circulated biopolymeric nanocarriers. *Bioconjugate Chemistry* **2017**, *28*, 1351-1355.
34. Deng, H.; Zhao, X.; Deng, L.; Liu, J.; Dong, A. Reactive oxygen species activated nanoparticles with tumor acidity internalization for precise anticancer therapy. *Journal of Controlled Release* **2017**, *255*, 142-153.
35. Shalgunov, V.; Zaytseva-Zotova, D.; Zintchenko, A.; Levada, T.; Shilov, Y.; Andreyev, D.; Dzhumashev, D.; Metelkin, E.; Urusova, A.; Demin, O., *et al.* Comprehensive study of the drug delivery properties of poly(l-lactide)-poly(ethylene glycol) nanoparticles in rats and tumor-bearing mice. *Journal of Controlled Release* **2017**, *261*, 31-42.
36. Logie, J.; Ganesh, A.N.; Aman, A.M.; Al-awar, R.S.; Shoichet, M.S. Preclinical evaluation of taxane-binding peptide-modified polymeric micelles loaded with docetaxel in an orthotopic breast cancer mouse model. *Biomaterials* **2017**, *123*, 39-47.
37. Hou, J.; Guo, C.; Shi, Y.; Liu, E.; Dong, W.; Yu, B.; Liu, S.; Gong, J. A novel high drug loading mussel-inspired polydopamine hybrid nanoparticle as a ph-sensitive vehicle for drug delivery. *International Journal of Pharmaceutics* **2017**, *533*, 73-83.
38. Wang, L.; Li, D.; Hao, Y.; Niu, M.; Hu, Y.; Zhao, H.; Chang, J.; Zhang, Z.; Zhang, Y. Gold nanorod-based poly(lactic-co-glycolic acid) with manganese dioxide core-shell structured multifunctional nanoplatform for cancer theranostic applications. *International journal of nanomedicine* **2017**, *12*, 3059-3075.
39. He, R.; Yin, C. Trimethyl chitosan based conjugates for oral and intravenous delivery of paclitaxel. *Acta Biomaterialia* **2017**, *53*, 355-366.

40. Yan, G.; Wang, J.; Hu, L.; Wang, X.; Yang, G.; Fu, S.; Cheng, X.; Zhang, P.; Tang, R. Stepwise targeted drug delivery to liver cancer cells for enhanced therapeutic efficacy by galactose-grafted, ultra-ph-sensitive micelles. *Acta Biomaterialia* **2017**, *51*, 363-373.
41. Ansari, L.; Jaafari, M.R.; Bastami, T.R.; Malaekeh-Nikouei, B. Improved anticancer efficacy of epirubicin by magnetic mesoporous silica nanoparticles: In vitro and in vivo studies. *Artificial Cells, Nanomedicine, and Biotechnology* **2018**, *46*, 594-606.
42. Duan, D.; Wang, A.; Ni, L.; Zhang, L.; Yan, X.; Jiang, Y.; Mu, H.; Wu, Z.; Sun, K.; Li, Y. Trastuzumab- and fab' fragment-modified curcumin peg-plga nanoparticles: Preparation and evaluation in vitro and in vivo. *International journal of nanomedicine* **2018**, *13*, 1831-1840.
43. Wu, F.; Zhang, M.; Lu, H.; Liang, D.; Huang, Y.; Xia, Y.; Hu, Y.; Hu, S.; Wang, J.; Yi, X., *et al.* Triple stimuli-responsive magnetic hollow porous carbon-based nanodrug delivery system for magnetic resonance imaging-guided synergistic photothermal/chemotherapy of cancer. *ACS Applied Materials & Interfaces* **2018**, *10*, 21939-21949.
44. Park, J.; Park, J.E.; Hedrick, V.E.; Wood, K.V.; Bonham, C.; Lee, W.; Yeo, Y. A comparative in vivo study of albumin-coated paclitaxel nanocrystals and abraxane. *Small* **2018**, *14*, e1703670-e1703670.
45. Cheng, X.; Li, D.; Lin, A.; Xu, J.; Wu, L.; Gu, H.; Huang, Z.; Liu, J.; Zhang, Y.; Yin, X. Fabrication of multifunctional triple-responsive platform based on cus-capped periodic mesoporous organosilica nanoparticles for chemo-photothermal therapy. *International journal of nanomedicine* **2018**, *13*, 3661-3677.
46. Chai, S.; Kan, S.; Sun, R.; Zhou, R.; Sun, Y.; Chen, W.; Yu, B. Fabricating polydopamine-coated mose2-wrapped hollow mesoporous silica nanoplatform for controlled drug release and chemo-photothermal therapy. *International journal of nanomedicine* **2018**, *13*, 7607-7621.
47. Zhang, Y.; Guo, Z.; Cao, Z.; Zhou, W.; Zhang, Y.; Chen, Q.; Lu, Y.; Chen, X.; Guo, Q.; Li, C., *et al.* Endogenous albumin-mediated delivery of redox-responsive paclitaxel-loaded micelles for targeted cancer therapy. *Biomaterials* **2018**, *183*, 243-257.
48. Srimathveeravalli, G.; Abdel-Atti, D.; Pérez-Medina, C.; Takaki, H.; Solomon, S.B.; Mulder, W.J.M.; Reiner, T. Reversible electroporation–mediated liposomal doxorubicin delivery to tumors can be monitored with 89zr-labeled reporter nanoparticles. *Molecular Imaging* **2018**, *17*, 1536012117749726.
49. Xu, C.; Chen, F.; Valdovinos, H.F.; Jiang, D.; Goel, S.; Yu, B.; Sun, H.; Barnhart, T.E.; Moon, J.J.; Cai, W. Bacteria-like mesoporous silica-coated gold nanorods for positron emission tomography and photoacoustic imaging-guided chemo-photothermal combined therapy. *Biomaterials* **2018**, *165*, 56-65.
50. Yu, G.; Yang, Z.; Fu, X.; Yung, B.C.; Yang, J.; Mao, Z.; Shao, L.; Hua, B.; Liu, Y.; Zhang, F., *et al.* Polyrotaxane-based supramolecular theranostics. *Nature Communications* **2018**, *9*, 766.
51. Motamarry, A.; Negussie, A.H.; Rossmann, C.; Small, J.; Wolfe, A.M.; Wood, B.J.; Haemmerich, D. Real-time fluorescence imaging for visualization and drug uptake prediction during drug delivery by thermosensitive liposomes. *International Journal of Hyperthermia* **2019**, *36*, 817-826.
52. Dunne, M.; Epp-Ducharme, B.; Sofias, A.M.; Regenold, M.; Dubins, D.N.; Allen, C. Heat-activated drug delivery increases tumor accumulation of synergistic chemotherapies. *Journal of Controlled Release* **2019**, *308*, 197-208.

53. Šimek, M.; Hermannová, M.; Šmejkalová, D.; Foglová, T.; Souček, K.; Binó, L.; Velebný, V. Lc–ms/ms study of in vivo fate of hyaluronan polymeric micelles carrying doxorubicin. *Carbohydrate Polymers* **2019**, *209*, 181-189.
54. Duan, X.; Chan, C.; Han, W.; Guo, N.; Weichselbaum, R.R.; Lin, W. Immunostimulatory nanomedicines synergize with checkpoint blockade immunotherapy to eradicate colorectal tumors. *Nature Communications* **2019**, *10*, 1899.
55. Wang, Y.; Liu, H.; Yao, D.; Li, J.; Yang, S.; Zhang, C.; Chen, W.; Wang, D. 18f-labeled magnetic nanoparticles for monitoring anti-angiogenic therapeutic effects in breast cancer xenografts. *Journal of Nanobiotechnology* **2019**, *17*, 105.
56. Mukerabigwi, J.F.; Yin, W.; Zha, Z.; Ke, W.; Wang, Y.; Chen, W.; Japir, A.A.-W.M.M.; Wang, Y.; Ge, Z. Polymersome nanoreactors with tumor ph-triggered selective membrane permeability for prodrug delivery, activation, and combined oxidation-chemotherapy. *Journal of Controlled Release* **2019**, *303*, 209-222.
57. Zhang, L.; Su, H.; Wang, H.; Li, Q.; Li, X.; Zhou, C.; Xu, J.; Chai, Y.; Liang, X.; Xiong, L., *et al.* Tumor chemo-radiotherapy with rod-shaped and spherical gold nano probes: Shape and active targeting both matter. *Theranostics* **2019**, *9*, 1893-1908.
58. Jadon, R.S.; Sharma, M. Docetaxel-loaded lipid-polymer hybrid nanoparticles for breast cancer therapeutics. *Journal of Drug Delivery Science and Technology* **2019**, *51*, 475-484.
59. Parker, C.L.; McSweeney, M.D.; Lucas, A.T.; Jacobs, T.M.; Wadsworth, D.; Zamboni, W.C.; Lai, S.K. Pretargeted delivery of peg-coated drug carriers to breast tumors using multivalent, bispecific antibody against polyethylene glycol and her2. *Nanomedicine: Nanotechnology, Biology and Medicine* **2019**, *21*, 102076.
60. Zhang, Y.; Liu, Y.; Gao, X.; Li, X.; Niu, X.; Yuan, Z.; Wang, W. Near-infrared-light induced nanoparticles with enhanced tumor tissue penetration and intelligent drug release. *Acta Biomaterialia* **2019**, *90*, 314-323.
61. Mondal, L.; Mukherjee, B.; Das, K.; Bhattacharya, S.; Dutta, D.; Chakraborty, S.; Pal, M.M.; Gaonkar, R.H.; Debnath, M.C. Cd-340 functionalized doxorubicin-loaded nanoparticle induces apoptosis and reduces tumor volume along with drug-related cardiotoxicity in mice. *International journal of nanomedicine* **2019**, *14*, 8073-8094.
62. Mu, J.; Zhong, H.; Zou, H.; Liu, T.; Yu, N.; Zhang, X.; Xu, Z.; Chen, Z.; Guo, S. Acid-sensitive pegylated paclitaxel prodrug nanoparticles for cancer therapy: Effect of peg length on antitumor efficacy. *Journal of Controlled Release* **2020**, *326*, 265-275.
63. Kazi, J.; Sen, R.; Ganguly, S.; Jha, T.; Ganguly, S.; Chatterjee Debnath, M. Folate decorated epigallocatechin-3-gallate (egcg) loaded plga nanoparticles; in-vitro and in-vivo targeting efficacy against mda-mb-231 tumor xenograft. *International Journal of Pharmaceutics* **2020**, *585*, 119449.
64. Ding, Y.; Sun, Z.; Tong, Z.; Zhang, S.; Min, J.; Xu, Q.; Zhou, L.; Mao, Z.; Xia, H.; Wang, W. Tumor microenvironment-responsive multifunctional peptide coated ultrasmall gold nanoparticles and their application in cancer radiotherapy. *Theranostics* **2020**, *10*, 5195-5208.
65. Xie, B.; Wan, J.; Chen, X.; Han, W.; Wang, H. Preclinical evaluation of a cabazitaxel prodrug using nanoparticle delivery for the treatment of taxane-resistant malignancies. *Molecular Cancer Therapeutics* **2020**, *19*, 822-834.

66. Bort, G.; Lux, F.; Dufort, S.; Crémillieux, Y.; Verry, C.; Tillement, O. Epr-mediated tumor targeting using ultrasmall-hybrid nanoparticles: From animal to human with theranostic aguix nanoparticles. *Theranostics* **2020**, *10*, 1319-1331.
67. Zhou, Z.; Zhang, Q.; Yang, R.; Wu, H.; Zhang, M.; Qian, C.; Chen, X.; Sun, M. Atp-charged nanoclusters enable intracellular protein delivery and activity modulation for cancer theranostics. *iScience* **2020**, *23*, 100872.
68. Guo, J.; Yu, Z.; Das, M.; Huang, L. Nano codelivery of oxaliplatin and folinic acid achieves synergistic chemo-immunotherapy with 5-fluorouracil for colorectal cancer and liver metastasis. *ACS Nano* **2020**, *14*, 5075-5089.
69. Sofias, A.M.; Toner, Y.C.; Meerwaldt, A.E.; van Leent, M.M.T.; Soultanidis, G.; Elschot, M.; Gonai, H.; Grendstad, K.; Flobak, Å.; Neckmann, U., *et al.* Tumor targeting by αvβ3-integrin-specific lipid nanoparticles occurs via phagocyte hitchhiking. *ACS Nano* **2020**, *14*, 7832-7846.
70. Hao, Q.; Wang, Z.; Zhao, W.; Wen, L.; Wang, W.; Lu, S.; Xing, D.; Zhan, M.; Hu, X. Dual-responsive polyprodrug nanoparticles with cascade-enhanced magnetic resonance signals for deep-penetration drug release in tumor therapy. *ACS Applied Materials & Interfaces* **2020**, *12*, 49489-49501.
71. Cong, Z.; Zhang, L.; Ma, S.-Q.; Lam, K.S.; Yang, F.-F.; Liao, Y.-H. Size-transformable hyaluronan stacked self-assembling peptide nanoparticles for improved transcellular tumor penetration and photo–chemo combination therapy. *ACS Nano* **2020**, *14*, 1958-1970.
72. Owen, J.; Thomas, E.; Menon, J.; Gray, M.; Skaripa-Koukelli, I.; Gill, M.R.; Wallington, S.; Miller, R.L.; Vallis, K.A.; Carlisle, R. Indium-111 labelling of liposomal hegf for radionuclide delivery via ultrasound-induced cavitation. *Journal of Controlled Release* **2020**, *319*, 222-233.
73. Xiong, X.; Xu, Z.; Huang, H.; Wang, Y.; Zhao, J.; Guo, X.; Zhou, S. A nir light triggered disintegratable nanoplatform for enhanced penetration and chemotherapy in deep tumor tissues. *Biomaterials* **2020**, *245*, 119840.
74. Katifelis, H.; Mukha, I.; Bouziotis, P.; Vityuk, N.; Tsoukalas, C.; Lazaris, A.C.; Lyberopoulou, A.; Theodoropoulos, G.E.; Efstathopoulos, E.P.; Gazouli, M. Ag/au bimetallic nanoparticles inhibit tumor growth and prevent metastasis in a mouse model. *International journal of nanomedicine* **2020**, *15*, 6019-6032.
75. Lu, T.; Haemmerich, D.; Liu, H.; Seynhaeve, A.L.B.; van Rhoon, G.C.; Houtsmuller, A.B.; ten Hagen, T.L.M. Externally triggered smart drug delivery system encapsulating idarubicin shows superior kinetics and enhances tumoral drug uptake and response. *Theranostics* **2021**, *11*, 5700-5712.
76. Liping, Y.; Jian, H.; Zhenchao, T.; Yan, Z.; Jing, Y.; Yangyang, Z.; Jing, G.; Liting, Q. Gsh-responsive poly-resveratrol based nanoparticles for effective drug delivery and reversing multidrug resistance. *Drug Delivery* **2022**, *29*, 229-237.
77. Nabi, P.N.; Vahidfar, N.; Tohidkia, M.R.; Hamidi, A.A.; Omidi, Y.; Aghanejad, A. Mucin-1 conjugated polyamidoamine-based nanoparticles for image-guided delivery of gefitinib to breast cancer. *International Journal of Biological Macromolecules* **2021**, *174*, 185-197.
78. Cvjetinović, Đ.; Prijović, Ž.; Janković, D.; Radović, M.; Mirković, M.; Milanović, Z.; Mojović, M.; Škalamera, Đ.; Vranješ-Đurić, S. Bioevaluation of glucose-modified liposomes as a

potential drug delivery system for cancer treatment using 177-lu radiotracking. *Journal of Controlled Release* **2021**, *332*, 301-311.
79. El-Safoury, D.M.; Ibrahim, A.B.; El-Setouhy, D.A.; Khowessah, O.M.; Motaleb, M.A.; Sakr, T.M. Amelioration of tumor targeting and in vivo biodistribution of 99mtc-methotrexate-gold nanoparticles (99mtc-mex-aunps). *Journal of Pharmaceutical Sciences* **2021**, *110*, 2955-2965.
80. Parakhonskiy, B.V.; Shilyagina, N.Y.; Gusliakova, O.I.; Volovetskiy, A.B.; Kostyuk, A.B.; Balalaeva, I.V.; Klapshina, L.G.; Lermontova, S.A.; Tolmachev, V.; Orlova, A., *et al.* A method of drug delivery to tumors based on rapidly biodegradable drug-loaded containers. *Applied Materials Today* **2021**, *25*, 101199.
81. El-Safoury, D.M.; Ibrahim, A.B.; El-Setouhy, D.A.; Khowessah, O.M.; Motaleb, M.A.; Sakr, T.M. Gold nanoparticles for 99mtc-doxorubicin delivery: Formulation, in vitro characterization, comparative studies in vivo stability and biodistribution. *Journal of Radioanalytical and Nuclear Chemistry* **2021**, *328*, 325-338.
82. Al-Jamal, W.T.; Kostarelos, K. Mild hyperthermia accelerates doxorubicin clearance from tumour-extravasated temperature-sensitive liposomes. *Nanotheranostics* **2022**, *6*, 230-242.
83. Kannaka, K.; Sano, K.; Munekane, M.; Yamasaki, T.; Hagimori, M.; Mukai, T. Enhanced therapeutic effect of liposomal doxorubicin via bio-orthogonal chemical reactions in tumors. *Molecular Pharmaceutics* **2022**, *19*, 1400-1409.
84. Han, Y.; Dong, Z.; Wang, C.; Li, Q.; Hao, Y.; Yang, Z.; Zhu, W.; Zhang, Y.; Liu, Z.; Feng, L. Ferrous ions doped calcium carbonate nanoparticles potentiate chemotherapy by inducing ferroptosis. *Journal of Controlled Release* **2022**, *348*, 346-356.
